# Once-daily dolutegravir/lamivudine fixed-dose formulations in children living with HIV: a pharmacokinetic and safety sub-study nested in the open-label, multicentre, randomised, non-inferiority D3/PENTA 21 trial

**DOI:** 10.1016/j.ebiom.2025.105929

**Published:** 2025-09-26

**Authors:** Lisanne A.H. Bevers, Gabriela Toledo, Muzamil Kisekka, Elizabeth Kaudha, Grace M. Ahimbisibwe, Isabelle Deprez, Tiyara Arumugan, Ebrahim Variava, Avy Violari, Iona White, Dickson Bbuye, Annet Nanduudu, Enoch Mulwanyi, Pauline Amuge, Diana A. Rutebarika, Adeodata Kekitiinwa, Cissy Kityo, Philippa Musoke, Moherndran Archary, Justine Boles, Margaret J. Thomason, Saskia N. de Wildt, Carlo Giaquinto, Angela Colbers, David M. Burger, Ann M. Buchanan, Man K. Chan, Tom G. Jacobs, Anna Turkova, D3 TRIAL TEAM, Carlo Giaquinto, Alessandra Nardone, Gabija Morkunaite, Anna Turkova, Debbie Ford, Man Chan, Gabriela Toledo, Elizabeth James, Mags Thomason, Nazia Parkar, Iona White, Anna Parker, Anas Omar, Zainab Alkurwi, Moira Spyer, Katja Doerholt, Stephen Townsend, Hannah Sweeney, Margaret Hook, Alvin Daramola-Rose, Lee Barker, Lu Gao, Matteo Quartagno, Diana Gibb, Tim R. Cressey, Suwalai Chalermpantmetagul, Rukchanok Peongjakta, Ketwarin Pongkawong, Worathip Sripaoraya, Warunee Khamjakkaew, Namthip Kruenual, Pra-ornsuda Sukrakanchana, Ampika Kaewbundit, Adeodata R. Kekitiinwa, Pauline Amuge, Christine Namugwanya, Lameck Kiyimba, Resty Babirye Okello, Florence Namuli, Ronald Nabimba, Angella Baita, Dickson Bbuye, Susan Tukamuhebwa, Rachael Namuddu Kikabi, Rose Jacqueline Kadhuba, Henry Balwa, Sarah Nabukalu, Muzamil Nsibuka Kisekka, Anthony Kirabira, Lekku Lawrence, Judith Tikabibamu, Maria Benita Aino, Gerald Agaba Muzorah, Collins Mujyanama, Annet Nalugo, Priscilla Namubiru, Cissy Kityo, Victor Musiime, Elizabeth Kaudha, Annet Nanduudu, Henry Mugerwa, Caroline Otike, Emmanuel Mujyambere, Dridah Nakiboneka, Barbara Mukanza, Julius Tumusiime, Onen Gilbert, Ritah Mbabazi, Abigail Atwine, Priscilla Kyobutungi, Juliet Ategeka, Alex Musiime, Sharif Musumba, Rashidah Nazzinda, Nicholas Wangwe, Phyllis Mwesigwa, Diana Rutebarika, Jameena Elsauko, Mangadalen Nansaigi, Mercy Tukamushaba, Alice Mulindwa, Aidah Nakalyango, Ocitti Paul, Christine Nambi, Milly Ndigendawani, Mariam Naabalamba, Eram David, Odochi Denis, Baliruno David, Ezra Lutalo, Eddie Rubanga, Josephine Namusanje, Josephine Kobusingye, Disan Mulima, Maria Nannungi, Faith Balmoi, Charles Draleku, Faith Mbasani, Crispus Katemba, Juliet Ankunda, Julian Tusiime, Benson Ouma Juma, Philippa Musoke, Grace Miriam Ahimbisibwe, Hajira Kataike, Winnie Nansamba, David Balamusani, Rosemary Namwanje, Enoch Mulwanyi, Gerald Bright Businge, Maxensia Owor, Aziida Nabukeera, Ruth Nakku, Zainab Nakivumbi Nassoma, Immaculate Nayiga Serunjogi, Barbara Musoke Nakirya, Sarah Nakabuye, Erinah Kyomukama, Rebecca Wampamba, Stella Nalusiba, Emmanuel Mayanja, Donald Wagaana, Zaam Zinda Nakawungu, Sarah Babirye Ssebabi, Olivia Higiro Kaboggoza, Edith Nabawubye, Mildred Kabasonga, Harriet Namusisi, Judith Nampewo, Agnes Mary Mugagga, Richard Isabirye, Francis Sserugo, Annet Kawuma, Agnes Namuddu, Joanita Nankya Baddokwaya, Juliet Nanyonjo, Winifred Kaahwa, Maria Musisi, Paula Mubiru Namayanja, Doreen Twenatwine, Robert Byuma, Winifred Luwedde, Margaret Mugenyi, Joseph Mutebo, Francis Katongole, Fabian Okello, Max Kiwewa, Ronald Okwera, Derick Balungi, Obed Tumwizere, Teopista Nakyanzi, Ann Kankindi, Maria Janine Nambusi, Ivan Rukundo, Henry Odyek, Barnabas Weere, Bosco Kafufu, David Ssebunya, Africano Kamugisha, Emmanual Hakizimana, Charles Nyende, Evelyn Akurut, Johnson Tumwesigye, Brenda Catherine Kakayi, Rebecca Sakwa, Mark Ssenyonga, Joseph Semakula, Joyce Mwebaza, Judith Kainza, Miscah Babirye Otim, Moherndran Archary, Rosie Mngqibisa, Tiyara Arumugam, Sundrapragasen Pillay, Raziya Bobat, Nombuso Nkosi, Nozibusiso Rejoice Mosia, Sheleika Singh, Shingirai Chimene, Jabu Mkhulise, Innocentia Thandokuhle Mncube, Zethu Mnyandu, Michelle Moodley, Popi Shabalala, Simangele Bengu, Ida Elizabeth Mundhree, Ebrahim Variava, Tumelo Moloantoa, Nadia Sabet, Ryan Sabet, Modiehi Mosala, Itumeleng Holele, Eva Mogotsi, Angelinah Montwedi, Serame Mokoena, Keabetswe Kotsokoane, Dineo Rampai, Avy Violari, Lerato Maretlwa, Palesa Tshipunyane, Iris Matotong, Abraham Mammwn Pattamukkil, Nkazimulo Xulu, Sthembiso Mhlanga, Ofhani Harmious Makhari, Gifty Okyere Manu, Zukisa Mpeluza, Tumelo Moloantoa, Mbusiseni Ngema, Abdul Kaka, Rieta Stokes, Nadia Bellingan, Linique Le Grange, Leoni Stytler, Zakkiyya Jeeva, Avy Violari, Afaaf Liberty, Mandisa Nyati, Haseena Cassim, Sisinyana Ruth Mathiba, Lindiwe Maseko, Precious Ndebele, Jackie Brown, Emily Lebotsa, Deirdre Josipovic, Mantwa Kunene, Tryphinah Madonsela, Nasreen Abrahams, Zaakirah Essack, Zandisile Mtshali, Dipuo Dhlomoza, Thabile Degracia Hlomuka, Valerie Khemese, Pradthana Ounchanum, Suchada Ruenglerdpong, Areerat Kongponoi, Kanyanee Kaewmamueng, Warunee Srisuk, Yupawan Thaweesombat, Sukanda Denjanta, Jutarat Thewsoongnoen, Naowarat Kunyanone, Sakulrat Srirojana, Doungjai Donngern, Petcharat Phunkhum, Arisara Kamkoonmongkol, Thananya Naksomboon, Nonthaporn Na Kalasin, Ussanee Srirompotong, Athiporn Rungsapphaiboon, Wallapa Daechasatain, Wanchalerm Boonsub, Patamawadee Sudsaard, Thunyasiri Dechboran, Manthana Mitchai, Thanawat Samranphit, Orapin Wannasri, Kriangkrai Kongsuk, Suparat Kanjanavanit, Rujirek Kamolrattana, Thannapat Chankun, Jiraporn Punyayen, Chayakorn Saewtrakool, Pacharaporn Yingyong, Raungwit Junkaew, Benjawan Thomyota, Kanokkorn Sawasdichai, Chaiwat Ngampiyaskul, Nantika Paiboon, Benjama I-nala, Wanna Chamjamrat, Pisut Greetanukroh, Chanthaporn Imbumroong, Sasipass Khannak, Rattana Chahmeanprabnakorn, Steven Welch, Ezgi Seager, Yvonne Beuvink, Melanie Rooney, Laura Thrasyvoulou, Katie Price, George Gavin, Sue Fagg, Baldip Kaur, Molly Williams, Anna Turkova, Alasdair Bamford, Delane Shingadia, Jade Sugars, Olamide Alimi, Carolyn Chan, Kelly Cripps, Sabina Zahed, John Koroma, Clàudia Fortuny, Antoni Noguera-Julian, Anna Vazquez Marchan, Cristina López Hidalgo, Sílvia Cuadras Ferrando, Alba Murciano, Miriam Coto, Kenia Sánchez, Bonaventura Ruiz, Pablo Rojo, Cristina Epalza, Luis Prieto, Jose Tomas Ramos, Ianire Gallego, Verónica Martín, Lilit Manukyan, Manuel Gijon, David Burger, Angela Colbers, Tom Jacobs, Lisanne Bevers, Dan Carr, Andrew Owen, Rebecca Jensen, Eleni Nastouli, Moira Spyer, Matt Byott, Ian Botha, Paul Revill, Simon Walker, Magda Conway, Lungile Jafta, Mercy Shibemba, Hermione Lyall, Elizabeth Maleche Obimbo, Theodore Ruel, Alex Compagnucci, Imelda Mahaka, Fanele Bulose, Gugulethu Bomela, Anna Turkova, Cissy Kityo, Tim Cressey, Avy Violari, Anton Pozniak, Jane Crawley, Rodolphe Thiébaut, Helen McIlleron

**Affiliations:** a Department of Pharmacy, Pharmacology and Toxicology, Radboudumc Research Institute for Medical Innovation (RIMI), Radboud University Medical Center, Nijmegen, the Netherlands; b Medical Research Council Clinical Trials Unit at University College London, UK; c Baylor College of Medicine Children's Foundation, Kampala, Uganda; d Joint Clinical Research Centre, Kampala, Uganda; e Makerere University-Johns Hopkins University Research Collaboration, Kampala, Uganda; f GlaxoSmithKline, Mississauga, Canada; g Certara, Princeton, NJ, USA; h Department of Paediatrics and Children Health, King Edward VIII Hospital, Enhancing Care Foundation, University of KwaZulu-Natal, Durban, South Africa; i Perinatal HIV Research Unit, University of the Witwarsrand, Johannesburg, South Africa; j Department of Paediatrics and Child Health, Makerere University College of Health Sciences, Kampala, Uganda; k ViiV Healthcare, London, UK; l Department of Intensive Care, Radboud University Medical Center, Nijmegen, the Netherlands; m Department of Neonatal and Pediatric Intensive Care, Erasmus MC Sophia Children's Hospital Rotterdam, the Netherlands; n Department of Women and Child Health, Fondazione Penta ETS, University of Padova, Padova, Italy; o ViiV Healthcare, Durham, NC, USA

**Keywords:** Dolutegravir, Lamivudine, Paediatric, HIV, Fixed-dose formulation, Two-drug regimen

## Abstract

**Background:**

Two-drug regimen dolutegravir/lamivudine (DTG/3TC) is recommended as an alternative to standard three-drug regimens in adult treatment guidelines. This nested pharmacokinetic sub-study within the D3/Penta 21 randomised trial (#NCT04337450) assessed DTG and 3TC concentrations and safety in virologically-suppressed children, switching to once-daily DTG/3TC fixed-dose formulations.

**Methods:**

Children aged 2–<15 years received either 5/30 mg DTG/3TC dispersible tablets (DT) or 50/300 mg film-coated tablets (FCT), using WHO weight band (WB)-aligned dosing: 10–<14 kg 4 DTs; 14–<20 kg 5 DTs; 20–<25 kg 6 DTs or 1 FCT; 25–<40 kg 1 FCT. A minimum of 8-evaluable pharmacokinetic curves per WB/formulation were targeted for 24 h pharmacokinetic profiling (t = 0, 1, 2, 3, 4, 6, and 24 h post-dosing) at steady state. The number of children with DTG C_trough_ <0.32 mg/L (EC90) and <0.064 mg/L (PA-IC90) were summarised. Safety was evaluated through 48 weeks in eligible children consented to the pharmacokinetic sub-study.

**Findings:**

Between 11th May 2022 and 31st May 2023, 82 children consented for the sub-study. Seventy-two were included in the pharmacokinetic analysis; median (IQR) age was 7.1 (4.9–10.0) years and weight 21.6 (17.7–24.8) kg. DTG geometric mean (GM) (%CV) C_trough_ and AUC_0–24 h_ were 0.82 (54) mg/L and 66.2 (35) h∗mg/L. 3TC GM-AUC_0–24 h_ was 16.2 (45) h∗mg/L. Three children had DTG C_trough_<0.32 mg/L, all had DTG C_trough_ ≥0.064 mg/L. In children weighing 20–<25 kg WB and taking 1 FCT (50/300 mg) 3TC GM AUC_0–24 h_ was 19% higher than in children ≥25 kg (1 FCT). Of 82 children, 3 had 4 serious adverse events (SAEs) and 5 had 6 grade ≥3 adverse events (AEs). No AEs were related to DTG/3TC or resulted in treatment discontinuation. No 3TC-related AEs or laboratory abnormalities were observed in children taking FCT in the 20–<25 kg WB. PK parameters were comparable to historical paediatric data from ODYSSEY (DTG) and IMPAACT2019 (3TC) trials.

**Interpretation:**

The study demonstrated adequate DTG and 3TC exposures with reassuring safety profiles using WB-based dosing, supporting licencing applications for dispersible and film-coated DTG/3TC formulations for paediatric use.

**Funding:**

The D3/Penta 21 trial is sponsored by Fondazione Penta Onlus ETS (Penta) and funded by 10.13039/100010877ViiV Healthcare.

**Research in context**

**Evidence before this study**

Paediatric antiretroviral therapy (ART) optimisation and simplification is critical to improve adherence, minimise long-term toxicities and maintain high efficacy. In adults, ART simplification through dual ART regimens has been shown to offer benefits over traditional triple-drug regimens, including smaller tablet sizes, fewer drug–drug interactions, and a better safety profile. Hence, the dual ART regimen consisting of DTG/3TC has been recommended as a first-line treatment option for adults in several international HIV treatment guidelines. In children, particularly, DTG/3TC could offer additional advantages by reducing cumulative drug exposure, mitigating long-term toxicity risks and preserving other ART options for future use. We searched PubMed for clinical trials, pharmacokinetic or cohort studies of the DTG/3TC regimen in the paediatric populations using “(pediatric OR paediatric OR children OR infant OR adolescents) AND (DTG/3TC OR dolutegravir/lamivudine)” and relevant conference abstracts up to January, 2025, not including the presentation of the data in this paper at a previous conference. Only the DANCE trial was identified, which is an ongoing single arm trial evaluating DTG/3TC including ART-naïve adolescents older than 12 years. At 96 weeks 22/25 (88%) of adolescents in DANCE were suppressed and no new safety concerns were observed compared to the established safety profile in adults. Based on these findings and extrapolation consisting of PK exposure matching to adults and corresponding adult efficacy data, the adult fixed-dose formulation DTG/3TC 50/300 mg (Dovato®) was approved for adolescents aged ≥12 years (weighing ≥25 kg by the FDA and ≥40 kg by the EMA) for HIV treatment initiation and maintenance. However, no clinical studies on dual ART regimens have yet been conducted in children under 12 years of age.

**Added value of this study**

This study assessed the pharmacokinetics and 48-week safety outcomes of once-daily DTG/3TC, using a dispersible tablet (DT) fixed-dose combination (5/30 mg) or the adult film-coated tablet (FCT) in virologically suppressed children aged 2–<15 years and weighing less than 40 kg. Intensive 24 h pharmacokinetic profiling was conducted using doses aligned with WHO weight bands. The findings confirmed that DTG and 3TC exposures achieved with dispersible DTG/3TC are consistent with the prior paediatric data from studies using three-drug ART such as ODYSSEY (for DTG) and IMPAACT2019 (for 3TC).

This study also evaluated use of the DTG/3TC FCT (50/300 mg) in children weighing 20–<25 kg, comparing it with six DTs (30/180 mg). This approach deviated from the WHO weight band dosing for 3TC in this weight band to simplify treatment, by allowing a single-tablet regimen for children who are able to swallow tablets. As anticipated, the adult FCT resulted in higher 3TC exposure in the 20–<25 kg weight band, but this dose was not associated with an increased risk of adverse events or clinically-significant laboratory abnormalities. Overall, safety evaluations across all weight bands found no new safety signals attributable to DTG/3TC, supporting the use of these FDCs in this paediatric population.

**Implications of all the available evidence**

This study assessed pharmacokinetics and safety of a dual ART regimen consisting of DTG/3TC in virologically suppressed children living with HIV from 2 years of age. The nested pharmacokinetic sub-study, integrated within a larger trial, provides timely regulatory data, supporting the licencing of DTG/3TC dispersible and film-coated formulations for paediatric use and expediting access to this simplified regimen.

## Introduction

Globally, children living with HIV continue to lag behind adults in receiving timely diagnosis, initiating antiretroviral therapy (ART) and achieving virological suppression.^1^ While the UNAIDS target aims for 95% of individuals on ART to achieve virological suppression <1000 copies/mL by 2030, in 2023, only 84% of children compared to 94% of adults met this benchmark. This disparity is largely driven by suboptimal paediatric formulations, adherence challenges and poor retention in care.^1^

The World Health Organisation (WHO) recommends a three-drug regimen (3DR) for children, combining two nucleoside/nucleotide analogue reverse-transcriptase inhibitors (NRTIs) and an integrase strand transfer inhibitor (INSTI), with dolutegravir (DTG) as the preferred INSTI.^2^^,^^3^ A pragmatic WHO weight-band dosing approach is commonly applied in paediatric antiretroviral treatment to align dose increments with increasing body weight, thereby simplifying prescribing and administration. While the 3DRs are effective, life-long treatment presents challenges related to sustaining adherence and managing long-term toxicities associated with cumulative exposures.^4^ As a result, research priorities have shifted towards optimising treatment administration and safety and improving health-related quality of life, beyond survival and viral suppression.^5^

Treatment optimisation aims to simplify medication intake, improve tolerability and safety while maintaining high efficacy. A two-drug regimen (2DR) of DTG and lamivudine (3TC) has demonstrated robust efficacy in adult trials, showing non-inferiority to 3DR among virologically-suppressed and ART-naïve adults with some safety advantages, including improved bone and renal health compared to tenofovir disoproxil fumarate (TDF)-containing 3DRs, as well as more favourable lipid biomarkers.^6^, ^7^, ^8^, ^9^ DTG/3TC has been included in the US and European guidelines as a recommended option for treatment initiation or treatment switch in adults.^10^^,^^11^

For children, the DTG/3TC regimen offers a child-friendly formulation, currently being studied as either film-coated tablets (FCT) or dispersible tablets (DT) depending on weight and ability to swallow pills, and allows for dosing without food requirements.^12^ It has limited potential for drug–drug interactions and avoids toxicities associated with a second NRTI, such as abacavir, zidovudine, TDF or tenofovir alafenamide (TAF), preserving these drugs for future treatment lines if needed.^13^The regimen also eliminates the need for HLA-B∗5701 testing for abacavir hypersensitivity in settings where such testing is implemented. Furthermore, as children experience fast growth and pubertal and neurodevelopmental maturation, reducing cumulative drug exposure may provide long-term safety benefits.^14^^,^^15^

The adult fixed-dose combination of 50/300 mg DTG/3TC (Dovato®) is currently approved by the FDA and EMA for treatment initiation and maintenance in adults and adolescents aged 12 years and older, with weight cut-offs of ≥25 kg (FDA) and ≥40 kg (EMA) respectively.^16^ DANCE (NCT03682848), a single arm study demonstrated 88% suppression <50 copies/mL at 96 weeks in 22/25 ART-naïve adolescents weighing ≥25 kg at enrolment.^17^ However, no licenced DTG/3TC fixed-dose formulations are available for younger children.

DTG is rapidly absorbed, with peak plasma concentrations typically reached within 2–3 h after oral administration. It is highly protein bound (>98%) and primarily metabolised via UGT1A1, with minor contributions from CYP3A4. DTG has an elimination half-life of approximately 14 h, supporting once-daily dosing.^18^ Lamivudine, in contrast, is a hydrophilic compound with low plasma protein binding, primarily eliminated unchanged by renal excretion. Its half-life is shorter (5–7 h), but intracellular triphosphate levels support once-daily administration.^19^ Both drugs have well-characterised PK profiles in adults and children.

The D3/Penta 21 trial (#NCT04337450) is an ongoing randomised controlled trial (RCT) evaluating the efficacy and safety of DTG/3TC compared to a triple DTG-based regimen in maintaining HIV suppression in children aged 2–<15 years. The trial assesses both a new DT formulation and the existing FCT, with the latter being evaluated for use in younger children than currently licenced. This paper presents the results of a nested pharmacokinetic sub-study within the D3/Penta 21 trial, which aims to assess DTG and 3TC concentrations and safety in virologically suppressed children weighing 6–<40 kg using WHO weight band (WB) aligned dosing.

## Methods

### Study design and participants

The D3/Penta 21 trial is an open-label, phase III, 96-week non-inferiority RCT being conducted in Spain, South Africa, Thailand, Uganda and the United Kingdom.^20^ At enrolment, participants were children aged 2–<15 years, weighing 6 kg or more, who were virologically suppressed <50 copies/mL for at least 6 months, with no history of treatment failure. There were no restrictions on enrolment by sex, which was recorded as reported by participants or, for younger children, by their parent or carer. Following screening and enrolment, scheduled visits occurred at weeks 4, 12 and then every 12 weeks thereafter, with clinical and laboratory assessments performed as per study protocol. The D3/Penta 21 trial study design is described in detail elsewhere.^20^

### Ethics

This study is being carried out in accordance with the principles of GCP as laid down by the ICH topic E6 (R2), the Declaration of Helsinki 2013, applicable national regulations, and ethical standards of all research committees. This trial was approved by Research Ethics Committees, Institutional Review Boards, and by all required regulatory authorities in each of the participating countries. Uganda: Joint Clinical Research Centre Institutional Review Board/Research Ethics Committee (JC1720; 02-Dec-2021; National Drug Authority (013/NDA/DPS/01/2022; 06-Jan-2022). South Africa: University of Witwatersrand Human Research Ethics Committee (200808 B; 18-Nov-2021); Pharma-Ethics (200,823,525; 17- Nov-2021); South African Health Products Regulatory Authority (20,200,807; 13-Dec-2021). Participants are enrolled in the trial following informed consent provided be a parent or legal guardian. Where appropriate, children who can understand the study were also asked to provide assent. Adolescents who reach the age of consent during the trial are required to re-confirm their continued participation by signing the informed consent form. For participants eligible for the intensive PK substudies, additional consent is obtained from parents or legal guardians, and assent from children where applicable. Consent and assent are also obtained for the storage of the samples for analyses outlined in the protocol and patient information sheets, as well as for future research studies. Informed consent in the trial is taken by a site Principal Investigator or a trained member of the trial team who have been delegated this activity.

### Intensive pharmacokinetic sub-study

The sub-study was nested within the main D3/Penta 21 trial and conducted in the DTG/3TC arm only. Eligible participants were children weighing 6 to <40 kg randomised to the DTG/3TC arm at pharmacokinetic sites in Uganda and South Africa, including: the Durban International Clinical Research Site (Durban), Perinatal HIV Research Units (PHRU) in Matlosana and Soweto, Baylor College of Medicine Children's Foundation (Baylor), Joint Clinical Research Center, Kampala (JCRC) and Makerere University—John Hopkins University Research Collaboration (MU-JHU). The aim was to enrol at least eight children with evaluable pharmacokinetic curves per WHO WB and formulation, with two formulations tested in 20–<25 kg WB (Table 1). The 6–<10 kg WB was included to align with the main trial's inclusion criteria and to accommodate children who could be small-for-age. The pharmacokinetic variability (CV) observed in the ODYSSEY PK substudies^21^^,^^22^ was around 30% across weight bands. According to Wang and colleagues, a power of 80% is reached with eight individuals and a coefficient of variation of 31–35%.^23^ Additional exclusion criteria included symptoms or conditions that could affect pharmacokinetics (i.e., severe diarrhoea, vomiting, renal disease or liver disease or severe malnutrition, defined as weight-for-height z-score more than three standard deviations below the median WHO growth standards^24^ or the use of concomitant medications known to have interactions with DTG/3TC (D3-protocol.pdf). In addition to the exclusion criteria, children were eligible for the 24 h intensive PK visit only if they had initiated DTG at least 21 days prior and DTG/3TC at least 7 days prior enrolment and had adhered to protocol-defined fasting requirements. Participants (or caregivers) had to confirm that the child had taken the weight-appropriate dose of DTG/3TC once daily in the morning for the three days preceding the PK visit, had not taken the morning dose on the day of sampling (as it was to be administered by study staff), and had not missed any doses in the preceding three days. All children were required to arrive for their first PK sample 20–30 h after the last DTG/3TC dose.

**Table 1:** D3/Penta 21 DTG/3TC fixed-dose combination dosing by weight band.

Weight band	5/30 mg DT DTG/3TC	50/300 mg FCT DTG/3TC
6–<10 kg	Three tablets (15/90 mg DT)	
10–<14 kg	Four tablets (20/120 mg DT)	
14–<20 kg	Five tablets (25/150 mg DT)	
20–<25 kg	Six tablets (30/180 mg DT)^a^	One tablet (50/300 mg FCT)^b^
25–<40 kg		One tablet (50/300 mg FCT)

a For children 20–<25 kg, WHO-recommended 3TC dose is 180 mg, EMA and FDA-recommended 3TC dose is 225 mg.

b The 3TC dose of 300 mg in children weighing 20–<25 kg and taking 50/300 mg FCT exceeds the currently recommended dose; participants in non-pharmacokinetic sites receive 50/300 mg FCT only.

Caregivers provided informed consent for their children's participation in the pharmacokinetic sub-study at screening, randomisation or follow-up in the main trial. Children who were old enough to understand their participation in the study provided informed assent. Eligible participants were invited to attend a 24 h intensive PK visit unless they were randomised to the control arm (after consenting/assenting prior to randomisation) or the applicable WB was closed to recruitment. Before initiating the intensive pharmacokinetic sampling, verbal consent and assent were reconfirmed.

All participants received either the paediatric fixed-dose DTG/3TC 5/30 mg DT or the adult fixed-dose 50/300 mg FCT, once daily dosed using WHO weight bands (Table 1). Children in the 20–<25 kg WB received either six 5/30 mg DTs or one 50/300 mg FCT depending on the formulation assigned to their site: DTs at Baylor and MU-JHU and FCTs at JCRC, Durban and PHRU. Dosing followed WHO recommendations for DTG and 3TC, except for children in the 20–<25 kg WB receiving the 50/300 mg FCT, which delivers a higher 3TC dose than currently recommended by EMA and FDA (225 mg) or WHO (180 mg). This dose was selected based on the well-established safety profile of 3TC in children^25^ and to simplify treatment by using a single adult FCT instead of multiple paediatric dispersible DTs.

All children who were eligible for the main trial, randomised to DTG/3TC and provided consent to the PK substudy were included in the safety population. Those who completed the intensive pharmacokinetic assessment with an evaluable pharmacokinetic curve were included in the intensive pharmacokinetic population.

### Pharmacokinetic sampling

A 24 h pharmacokinetic assessment (t = 0, 1, 2, 3, 4, 6, and 24 h post-dosing) was undertaken at least 21 days after initiating DTG to allow for a wash-out of pre-switch antiretrovirals that could influence DTG concentrations,^26^ and at least seven days after switching to the current DTG/3TC formulation and dosage to ensure a steady state on the regimen and dose.

Before observed drug intake, children were preferably fasted overnight, or for at least 3 h pre-dose. Intake of concomitant medication was not recommended within the first 2 h after DTG/3TC intake. Blood volumes taken were within blood draw limits for children established for research purposes.^27^ If the intensive pharmacokinetic sampling visit was unsuccessful as deemed by the site investigator and/or the D3/Penta 21 trial team, participants repeated the intensive pharmacokinetic sampling on a different date.

All blood samples were processed at each site's laboratory within 7 h of sampling. During this time, the samples were stored in a refrigerator or at room temperature and protected from light, due to the light sensitivity of DTG. After centrifugation, plasma was separated and stored in light protecting amber-coloured cryovials at −80 °C until arrival at the laboratory of the Department of Pharmacy, Radboudumc, Nijmegen, Netherlands for quantification. DTG and 3TC concentrations were measured using a validated combined liquid chromatography tandem mass spectrometry bioanalytical quantification method with a 0.01 mg/L and 0.005 mg/L lower limit of quantification (LLOQ) for DTG and 3TC, respectively. The calibration range was 0.01–20 mg/L for DTG and 0.005–10 mg/L for 3TC. The assay was externally validated through both the International Interlaboratory Quality Control Program for Measurement of Antiretroviral Drugs in Plasma and the Clinical Pharmacology Quality Assurance Program.^28^^,^^29^ Validation data, including accuracy and precision, are presented in Table S7.

### Outcomes

Primary pharmacokinetic outcomes were the area under the concentration–time curve (AUC_0–24 h_) and 24 h concentrations (C_trough_) for DTG and AUC_0–24 h_ for 3TC. We used AUC_0–24 h_ as the primary pharmacokinetic parameter for both DTG and 3TC to assess and compare overall drug exposure. C_trough_ was also included for DTG, guided by in-vitro and in-vivo evidence showing a correlation between C_trough_ and therapeutic efficacy. Secondary outcomes included maximum concentration (C_max_), elimination half-life (T_1/2_), and total oral clearance (Cl/F) for DTG and 3TC, and 24 h concentrations (C_trough_) for 3TC. Safety outcomes included clinical and laboratory adverse events (AEs) of any grade. All reported AEs and post-baseline emergent abnormal laboratory results were assessed from the first DTG/3TC dose to the earliest of end of week 48 visit window (377 days) or permanent DTG/3TC discontinuation (safety reporting period).

### Statistics

Median DTG and 3TC plasma concentrations were plotted by nominal time points. Pharmacokinetic parameters of DTG and 3TC were calculated using non-compartmental analysis (NCA) in Phoenix WinNonlin 64 software (version 8.4) and reported as geometric mean (GM) with coefficient of variation (CV%). The AUC_0–24 h_ was calculated using the linear up-log down trapezoidal method and Cl/F by dividing dose by AUC. Individual pharmacokinetic curves were assessed for biological plausibility. A PK curve was considered biologically implausible if it showed inconsistent time–concentration profiles (e.g. rising concentrations beyond expected absorption windows), missing samples critical for AUC calculation, or unexplained analytical outliers. In such cases, an investigation into sample collection and processing was conducted. PK curves were excluded from analysis if deemed unevaluable.

The pharmacokinetic parameters were compared with those from previous representative paediatric studies on DTG and 3TC.^21^^,^^22^^,^^30^ DTG AUC_0–24 h_ and C_trough_ were compared with data from the ODYSSEY pharmacokinetic sub-studies, which assessed DTG dosing in children weighing 3 kg to less than 40 kg, while 3TC AUC_0–24 h_ was compared with the IMPAACT 2019 pharmacokinetic sub-study, a dose-finding study for ABC/DTG/3TC fixed-dose dispersible formulation in children weighing 6 to less than 25 kg. For comparison, the overall GM and CV% C_trough_ and AUC_0–24 h_ across the weight bands were calculated using available in-house ODYSSEY data (DTG); for 3TC these were calculated by performing a meta-analysis^31^ of published IMPAACT 3TC results reported by WB.^21^^,^^22^^,^^30^^,^^31^

In addition, we aimed to achieve GM values per WB that were comparable with the reference ranges used for paediatric approvals of dolutegravir (Tivicay®) and dispersible ABC/DTG/3TC (Triumeq®): DTG C_trough_ 0.70–2.26 mg/L (Tivicay®) and 0.67–2.97 mg/L (Triumeq®), DTG AUC_0–24 h_ 37–134 h∗mg/L (Tivicay®) 35.1–134 h∗mg/L (Triumeq®), and 3TC AUC_0–24 h_ 6.3–26.5 h∗mg/L (Triumeq®). For DTG, we also report the proportion of individuals with C_trough_ lower than 0.32 mg/L (90% effective concentration (EC90) in a 10-day monotherapy study)^32^ and below 0.064 mg/L (in-vitro protein-adjusted 90% inhibitory concentration (PA-IC90)^33^ by WB.

Safety analysis included children eligible for the main trial, randomised to DTG/3TC who provided consent to the PK substudy (safety population). Descriptive statistics were used to analyse child characteristics by WB and safety outcomes by current WB over the safety reporting period detailed above using StataNow/MP software (version 18.5).

### Role of funders

The D3/Penta 21 trial is funded by ViiV Healthcare and Fondazione Penta ETS, the trial sponsor, provided additional funding support to their employees. ViiV Healthcare and Penta Foundation reviewed the study design and provided input on the trial protocol and the manuscript.

## Results

**Fig. 1:**
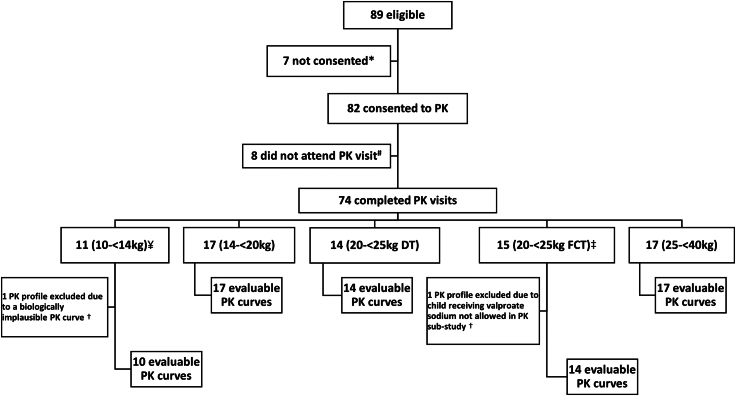
Disposition flowchart.

Between 11th May 2022 and 31st May 2023, 89 children were approached for consent to participate in the pharmacokinetic sub-study and consent was obtained for 82 (Table S1). Of these, intensive pharmacokinetic sampling was conducted for 74 children (Fig. 1). Eight children did not attend a pharmacokinetic visit due to their WB closing before the pharmacokinetic visit (n = 7) or scheduling issues (n = 1). Among the 74 children who completed pharmacokinetic sampling, one was excluded due to ineligibility (concurrent use of sodium valproate, which was not permitted), and another (10–<14 kg) was excluded for a biologically implausible PK curve, which showed rising concentrations from the second-to-last sampling timepoints instead of declining as expected during the elimination stage (Figure S3). Thus 72 children were included in the pharmacokinetic population (Table 2). All weighed between 10 and <40 kg, with evaluable curves distributed by weight band at pharmacokinetic visit as follows: 10 (10–<14 kg), 17 (14–<20 kg), 14 (20–<25 kg DT), 14 (20–<25 kg FCT), and 17 (25–<40 kg). Overall, 58% were female, with median age of 7.1 years [IQR: 4.9–10.0] and weight of 21.6 kg [IQR: 17.7–24.8]. All children were black-African, with most (86%) enrolled from Uganda. All adhered to overnight fasting (or at least 3 h pre-dose). DTG/3TC dosing typically occurred in the morning between 05:00 and 10:00 AM. None of the children were on any concomitant medications on the day of PK (except supplements and vitamins) that have significant drug interactions with DTG/3TC. Note that there were no children weighing 6–<10 kg enrolled in the main trial, hence no children in this WB were enrolled into the PK substudy.

**Table 2:** Child characteristics at intensive pharmacokinetic sampling day by weight band (N = 72).

Number of participants	Overall	10–<14 kg	14–<20 kg	20–<25 kg DT	20–<25 kg FCT	25–<40 kg
	N = 72	n = 10	n = 17	n = 14	n = 14	n = 17
Sex						
Male	30 (42%)	5 (50%)	8 (47%)	3 (21%)	7 (50%)	7 (41%)
Female	42 (58%)	5 (50%)	9 (53%)	11 (79%)	7 (50%)	10 (59%)
Race (black)	72 (100%)	10 (100%)	17 (100%)	14 (100%)	14 (100%)	17 (100%)
Age (years)						
Median (IQR)	7.1 (4.9, 10.0)	3.2 (2.4, 3.9)	5.0 (4.6, 6.1)	7.3 (7.0, 8.1)	8.1 (7.1, 9.1)	12.3 (10.8, 13.5)
Range	[2.3–13.8]	[2.3–4.5]	[3.7–7.0]	[6.6–11.4]	[5.5–11.1]	[7.2–13.8]
Weight (kg)						
Median (IQR)	21.6 (17.7, 24.8)	13.0 (12.6, 13.5)	17.9 (16.3, 19.0)	22.2 (21.3, 22.8)	22.6 (21.0, 24.0)	33.3 (29.4, 35.6)
Range	[12.5–39.5]	[12.5–14.1]	[14.1–19.8]	[20.5–23.1]	[20.0–25.1]	[26.7–39.5]
Weight-for-age^a^	53	10	17	12	12	2
Median (IQR)	−0.6 (−0.9, −0.1)	−0.8 (−1.5, −0.2)	−0.6 (−0.9, −0.3)	−0.5 (−0.8, −0.0)	−0.6 (−1.1, −0.1)	0.5 (−0.0, 1.0)
Range	[−2.2, −1.0]	[−2.2, −0.1]	[−1.5, −0.3]	[−1.1, −0.2]	[−2.2, −0.3]	[−0.0, −1.0]
3 to less than −2	2 (3%)	1 (10%)	0 (0%)	0 (0%)	1 (7%)	0 (0%)
2 to less than 0	43 (60%)	8 (80%)	15 (88%)	9 (64%)	10 (71%)	1 (6%)
0 or more	8 (11%)	1 (10%)	2 (12%)	3 (21%)	1 (7%)	1 (6%)
Missing	19 (26%)	0 (0%)	0 (0%)	2 (14%)	2 (14%)	15 (88%)
Height (cm)						
Median (IQR)	119 (108, 131)	90 (88, 95)	109 (103, 112)	119 (118, 122)	118 (117, 126)	142 (139, 148)
Range	[85–153]	[85–101]	[99–125]	[116–127]	[110–134]	[131–153]
Height-for-age^a^						
Median (IQR)	−1.0 (−1.6, −0.3)	−1.4 (−1.6, −1.1)	−0.4 (−1.5, 0.0)	−0.8 (−1.1, −0.3)	−0.8 (−1.6, −0.3)	−1.3 (−1.8, −0.1)
Range	[−4.1, −3.4]	[−3.6, −0.7]	[−2.6, −3.4]	[−4.0, −0.5]	[−4.1, −0.3]	[−2.6, −2.1]
Less than −3	3 (4%)	1 (10%)	0 (0%)	1 (7%)	1 (7%)	0 (0%)
3 to less than −2	6 (8%)	0 (0%)	1 (6%)	1 (7%)	1 (7%)	3 (18%)
2 to less than 0	52 (72%)	9 (90%)	11 (65%)	11 (79%)	11 (79%)	10 (59%)
0 or more	11 (15%)	0 (0%)	5 (29%)	1 (7%)	1 (7%)	4 (24%)
BMI (kg/m^2^)						
Median (IQR)	15.5 (14.6, 16.4)	15.9 (14.8, 16.5)	15.2 (13.9, 15.5)	15.1 (14.5, 16.0)	15.7 (14.6, 16.7)	16.1 (15.1, 17.2)
Range	[11.6–18.3]	[12.4–17.3]	[11.6–18.3]	[13.9–17.1]	[13.2–17.5]	[13.5–18.0]
BMI-for-age^a^						
Median (IQR)	−0.4 (−1.0, 0.2)	0.3 (−0.4, 0.8)	−0.0 (−1.0, 0.2)	−0.4 (−0.8, 0.4)	−0.1 (−0.6, 0.3)	−0.9 (−1.1, −0.5)
Range	[−3.5, −1.8]	[−2.3, −1.3]	[−3.5, −1.8]	[−1.7, −0.9]	[−2.2, −0.9]	[−3.3, −0.6]
Less than −3	2 (3%)	0 (0%)	1 (6%)	0 (0%)	0 (0%)	1 (6%)
3 to less than −2	5 (7%)	1 (10%)	1 (6%)	0 (0%)	2 (14%)	1 (6%)
2 to less than 0	41 (57%)	3 (30%)	8 (47%)	10 (71%)	7 (50%)	13 (76%)
0 or more	24 (33%)	6 (60%)	7 (41%)	4 (29%)	5 (36%)	2 (12%)
DTG exposure prior to D3 entry						
<1 month or no use	8 (11%)	1 (10%)	4 (24%)	0 (0%)	3 (21%)	0 (0%)
≥1 month	64 (89%)	9 (90%)	13 (76%)	14 (100%)	11 (79%)	17 (100%)
Country						
South Africa	10 (14%)	2 (20%)	3 (18%)	0 (0%)	5 (36%)	0 (0%)
Uganda	62 (86%)	8 (80%)	14 (82%)	14 (100%)	9 (64%)	17 (100%)

DT, dispersible tablet; FCT, film-coated tablet; IQR, interquartile range; BMI, body mass index; DTG, dolutegravir.

a WHO Child Growth Charts and WHO Reference 2007 Charts. Weight-for-age calculated for those younger than 10 years. If children were 10 years or older, weight for age will be missing.

**Fig. 2:**
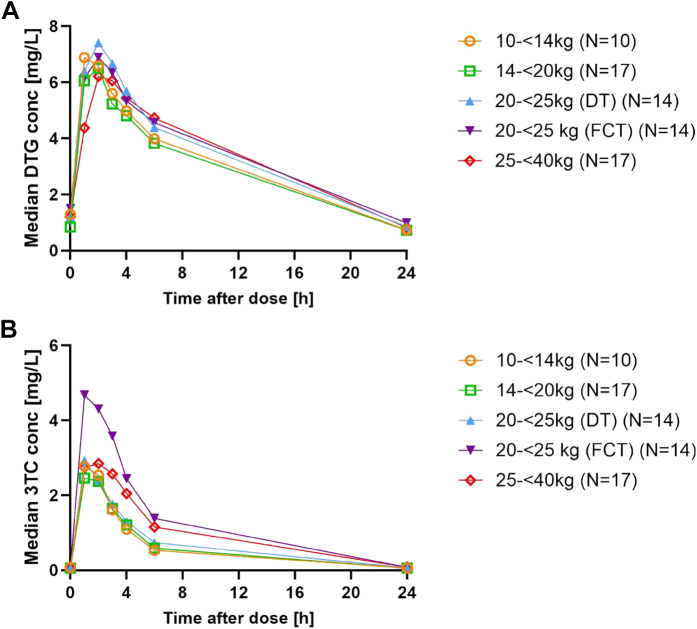
A: DTG median concentration time profile by weight band. B: 3TC median concentration time profile by weight band.

**Fig. 3:**
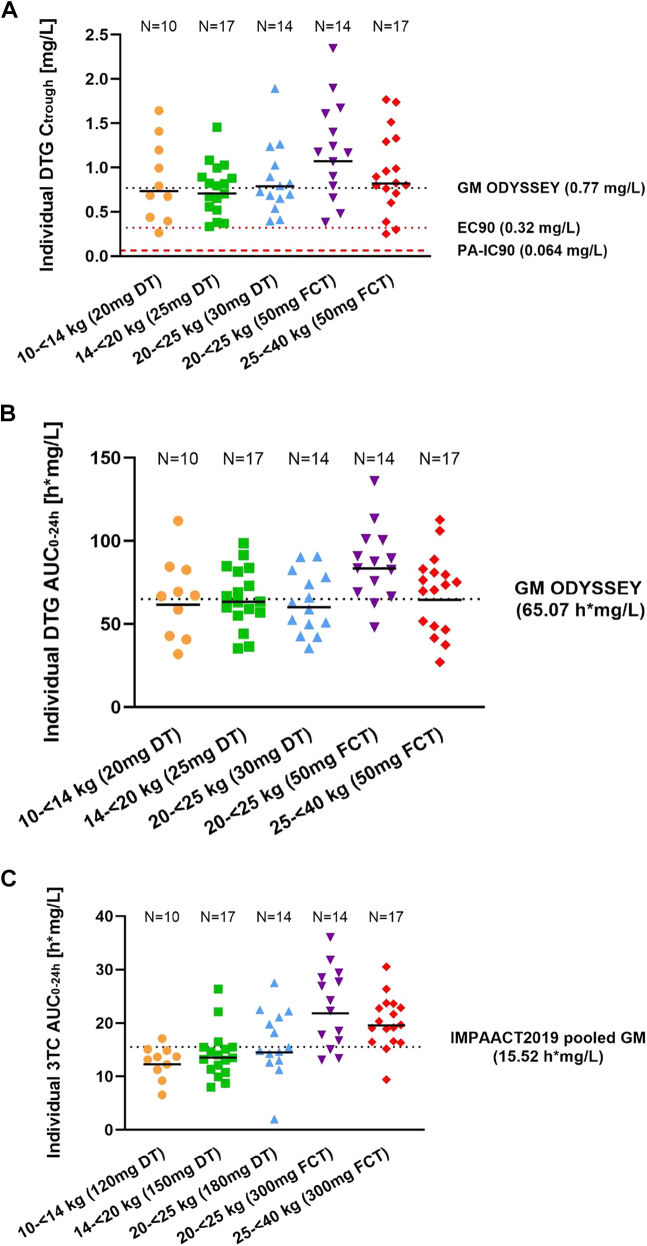
A: Individual DTG C_trough_ levels by weight band. The horizontal dotted black line indicates the GM C_trough_ level from ODYSSEY children weighing 10–<40 kg, calculated using available-in house ODYSSEY data. The dotted red line below shows the EC90 of 0.32 mg/L, and the lowest dashed red line is the PA-IC90 (0.064 mg/L). B: Individual DTG AUC_0–24 h_ levels by weight band. The horizontal dotted black line indicates the GM AUC_0–24 h_ level from ODYSSEY, calculated using available-inhouse ODYSSEY data. C: Individual 3TC AUC_0–24 h_ per weight band. The horizontal dotted black line indicates the pooled GM AUC_0–24 h_ level from IMPAACT2019, calculated by meta-analysis the reported GM values of the IMPAACT2019 study per WB.

For DTG, the overall GM (CV%) C_trough_ and AUC_0–24 h_ were 0.82 (54%) mg/L and 66.2 (35%) h∗mg/L (Table 3). In all WBs, DTG GM AUC_0–24 h_ and C_trough_ levels were within the ranges used in regulatory approvals of paediatric Tivicay® and Triumeq®. For 3TC, the overall GM (CV%) AUC_0–24 h_ was 16.2 h∗mg/L (45%) (Table 3). In all WBs, GM AUC_0–24 h_ levels for 3TC were within the range used for regulatory approvals of paediatric Triumeq®. In addition, DTG and 3TC concentrations were comparable with ODYSSEY and IMPAACT 2019, respectively. Ethnic composition in these three trials were comparable with all PK studies including a majority of participants of Black African ethnicity (100% in D3, 99% in ODYSSEY, and 65% in IMPAACT 2019) (Table S1). Median concentration time curves for DTG across individual WBs were overall similar across the weight bands in terms of shape, rate of decline, and total exposure (AUC; Fig. 2A and Figure S1). Among children weighing 20 kg–<25 kg, the GM AUC_0–24 h_ of 3TC was 23.37 h∗mg/L in those receiving the adult FCT and 13.56 h∗mg/L in those receiving the paediatric DT formulation (Figs. 2B and 3B and Figure S2). Only three children had a DTG C_trough_ level below the EC90 of 0.32 mg/L: one child in the 10–<14 kg WB, receiving DTG 20 mg (four DTs DTG/3TC 5/30 mg), had C_trough_ of 0.26 mg/L, and two children in the 25–<40 kg WB receiving the adult FCT (DTG/3TC 50/300 mg), had C_trough_ levels of 0.25 mg/L and 0.30 mg/L. All participants had C_trough_ levels above the PA-IC90 of 0.064 mg/L (Fig. 3A).

**Table 3:** Pharmacokinetic outcomes of DTG and 3TC within the D3/Penta 21 intensive PK study overall and by weight band.

	N	D3/Penta 21							ODYSSEY^21^^,^^22^	
		Dose by weight (mg/kg)	AUC_0–24 h_ (h∗mg/L)	C_max_ (mg/L)	C_trough_ (mg/L)	T_1/2_ (h)	T_max_ (h)	Cl/F (L/h)	AUC_0–24 h_ (h∗mg/L)	C_trough_ (mg/L)
**Dolutegravir (DTG)**										
Overall	72		66.25 (35)	6.49 (33)	0.82 (54)				65.07 (29)^b^	0.77 (54)^b^
10 kg–<14 kg	10	1.54 (1.42–1.60)	61.65 (40)	6.38 (30)	0.74 (64)	7.71 (16)	1.03 (1.00–2.00)	0.32 (40)	76.10 (21)	0.77 (57)
14 kg–<20 kg	17	1.40 (1.26–1.77)	63.48 (30)	6.68 (28)	0.70 (42)	7.30 (16)	2.00 (1.00–4.02)	0.39 (30)	69.93 (28)	0.87 (64)
20 kg–<25 kg (DT)	14	1.35 (1.30–1.46)	68.93 (32)	6.59 (45)	0.94 (38)	9.54 (72)	2.01 (0.00–4.03)	0.44 (32)	71.40 (26)	0.76 (73)
20 kg–<25 kg (FCT)	14	2.22 (1.99–2.50)	72.89 (35)	6.91 (28)	0.90 (66)	7.66 (23)	2.00 (1.00–6.00)	0.69 (35)	63.70 (26)	0.75 (44)
25 kg–<40 kg	17	1.50 (1.27–1.87)	64.53 (40)	6.00 (33)	0.82 (62)	7.86 (17)	2.00 (0.00–4.02)	0.77 (40)	25–<30 kg: 58.60 (28) 30–<40 kg: 53.50 (23)	25–<30 kg: 0.77 (43) 30–<40 kg: 0.63 (49)
		**D3/Penta 21**							**IMPAACT 2019**^30^	

**Lamivudine (3TC)**										
Overall	72		16.20 (45)	3.28 (50)	0.06 (37)				15.52 (21)^c^	0.06 (33)^c^
10 kg–<14 kg	10	9.23 (8.51–9.60)	12.29 (28)	2.88 (30)	0.06 (23)	5.22 (18)	1.01 (1.00–2.00)	9.77 (28)	14.20 (24)	0.05 (48)
14 kg–<20 kg	17	8.38 (7.58–10.64)	13.52 (31)	2.93 (39)	0.05 (36)	4.71 (18)	1.00 (1.00–4.02)	11.10 (31)	13.00 (16)	0.06 (37)
20 kg–<25 kg (DT)	14	8.13 (7.79–8.78)	13.56 (64)	2.81 (91)	0.06 (23)	6.01 (156)^a^	1.02 (0.00–3.02)	13.27 (64)	14.50 (17)	0.06 (18)
20 kg–<25 kg (FCT)	14	13.30 (11.95–15.00)	23.37 (30)	4.78 (24)	0.07 (41)	4.21 (15)	1.00 (1.00–2.00)	12.48 (30)	–	–
25 kg–<40 kg	17	9.01 (7.59–11.24)	19.55 (27)	3.29 (30)	0.07 (46)	4.27 (12)	1.02 (0.00–4.00)	15.35 (27)	21.70 (26)	0.08 (35)

Data presented as median (range) for dose by weight and T_max_ and geometric mean (percentage of coefficient variation) for other pharmacokinetic parameters. AUC_0–24 h_, area under the concentration–time over 24 h (dosing interval); C_max_, maximum concentration; C_trough_, minimum concentration; T_1/2_, elimination half-life; Cl/F, apparent oral clearance.

a This long T_1/2_ comes from one participant with an unusual flat curve.

b Calculated geometric means and percentage of coefficient variation from the available ODYSSEY pharmacokinetic data.

c Geometric means and percentage of coefficient variation from meta-analyses of published results by weight band.

There were five major protocol deviations in four of the 72 children relating to the intensive pharmacokinetic sampling (appendix Table S2). Two deviations occurred in two children in the 14–<20 kg WB: one child missed a single DTG/3TC dose two days prior to the pharmacokinetic visit and for another child, the DTG/3TC dose on the intensive pharmacokinetic sampling day was not properly dispersed according to guidance. Intensive pharmacokinetic sampling was repeated for these two participants, and data from their second successful visits were included in this analysis. The other three deviations involved missing blood samples at one or more time points for three children (2 in 14–<20 kg WB; 1 in 20–<25 kg FCT WB), these children were included in the analysis, and the missing values were extrapolated.

### Safety outcomes

Baseline characteristics of the 82 children included in the safety population are shown in Table S1. All children who completed pharmacokinetic sampling were on the per-protocol dose. Two children switched to ABC/3TC/DTG after their pharmacokinetic visit and before the end of the safety reporting period.

Of 82 participants, 3 (4%) experienced 4 serious adverse events requiring hospitalisation, and 5 (6%) had 6 grade ≥ 3 clinical AEs, including two participants with SAEs (Table 4, appendix Table S3). There were no deaths within the reporting period. Infections, primarily respiratory tract infections, were the most common AEs reported. All other AEs occurred in <5% of participants (appendix Tables S5). AE patterns were similar across weight bands. None of the reported events were deemed related to ART or resulted in treatment discontinuation or dose modification.

**Table 4:** Safety outcomes by current weight band and dose/formulation over 48-week follow-up (from first DTG/3TC dose to earliest of end of week 48 window or DTG/3TC discontinuation).

Dose and formulation	Overall	10–<14 kg	14–<20 kg	20–<25 kg	20–<25 kg	25–<40 kg
		(20/120 mg DT)	(25/150 mg DT)	(30/180 mg DT)	(50/300 mg FCT)	(50/300 mg FCT)
**Number of participants**	82	12	33	17	22	32
**Follow-up, weeks**^a^						
Median	53.9 (53.7, 53.9)	23.3 (11.8, 35.9)	39.1 (23.9, 53.9)	47.9 (35.7, 53.9)	42.0 (17.9, 53.9)	53.9 (30.6, 53.9)
Range	29.7–53.9	4.0–53.9	3.9–53.9	24.0–53.9	5.1–53.9	5.7–53.9
**Number of serious adverse events**^b^^,^^c^	4 [3]	0 [0]	1 [1]	0 [0]	3 [2]	0 [0]
Infections and infestations^a^	2 [2]	0 [0]	1 [1]	0 [0]	1 [1]	0 [0]
Respiratory, thoracic, and mediastinal disorders^b^	2 [2]	0 [0]	0 [0]	0 [0]	2 [2]	0 [0]
**Number of clinical events grade ≥3**	6 [5]	0 [0]	2 [2]	1 [1]	2 [1]	1 [1]
Blood lymphatic and system disorders^c^	2 [2]	0 [0]	0 [0]	1 [1]^d^	0 [0]	1 [1]
Gastrointestinal disorders^d^	1 [1]	0 [0]	1 [1]	0 [0]	0 [0]	0 [0]
Infections and infestations^a^	2 [2]	0 [0]	1 [1]	0 [0]	1 [1]	0 [0]
Respiratory, thoracic, and mediastinal disorders^e^	1 [1]	0 [0]	0 [0]	0 [0]	1 [1]	0 [0]
**Number of treatment emergent laboratory events grade ≥3**	5 [4]	1 [1]	3 [3]	1 [1]	0 [0]	0 [0]
Haemoglobin	1 [1]	0 [0]	1 [1]	0 [0]	0 [0]	0 [0]
Creatinine clearance	1 [1]	0 [0]	1 [1]^d^	0 [0]	0 [0]	0 [0]
Neutrophils	3 [3]	1 [1]	1 [1]	1 [1]^d^	0 [0]	0 [0]

Data are n [N]: number of events [number of participants]; MedDRA system organ classification shown.

MedDRA preferred terms: ^a^ cerebral malaria (14−<20 kg), pneumonia (20−<25 kg FCT); ^b^ cor pulmonale (20−<25 kg FCT), adenoidal hypertrophy (20−<25 kg FCT); ^c^ sickle cell anaemia with crisis (≥25 kg), febrile neutropenia (20−<25 kg DT); ^d^ diarrhoea (14−<20 kg); ^e^ cor pulmonale (20−<25 kg FCT).

a Follow-up based on time-updated DTG/3TC dose: dose changes were primarily due to weight gain and transitioning into the next weight band. Two children switched to ABC/3TC/DTG prior to the end of week 48 follow-up (377 days) and were censored at date of DTG/3TC discontinuation in the safety analysis; one child was enrolled in 20–<25 kg DT WB and the other in 20–<25 kg WB FCT. There were no reportable clinical AEs or laboratory events for either child by the end of week 48 follow-up.

b One of the SAEs (adenoidal hypertrophy) was classified as Grade 2 in a child receiving 50/300 mg FCT in 20−<25 kg WB.

c One child experienced two SAEs (pneumonia and cor pulmonale) in the 20−<25 kg WB (50/300 mg FCT).

d One child experienced two laboratory grade 3 abnormal results in two different weight bands: grade 3 reduced creatinine clearance occurred in the 14–<20 kg WB (25/120 mg DT) and grade 3 decreased neutrophils occurred in 20–<25 kg WB (30/180 mg DT), the latter was deemed to be clinically significant and related to grade 3 febrile neutropenia, reported as adverse event.

Grade ≥3 laboratory abnormalities occurred in 4 (5%) children (Table 4, appendix Table S4). Most laboratory abnormalities were grade 1 in severity (appendix Table S6). The most frequent abnormalities were reduced creatinine clearance (15/113, 13%), elevated alkaline phosphatase (18/113, 16%) and raised lactate (26/97, 27%). A numerically higher frequency of grade 1–2 lipase elevations was observed in 20–<25 kg WB FCT (4/17, 24%) compared to ≤1 (0–4%) in other WBs. Participants with elevated lipase had no relevant clinical symptoms, and no cases of pancreatitis were reported.

## Discussion

This pharmacokinetic sub-study nested within the D3/Penta 21 trial is the first to assess drug concentrations and safety of once-daily fixed-dose DTG/3TC formulations in children aged 2–<15 years.

The study showed that the selected WB doses achieved the targeted DTG and 3TC exposures. Primary pharmacokinetic outcomes (GM C_trough_ and AUC_0–24 h_ for DTG and GM AUC_0–24 h_ 3TC) were consistent with historical paediatric data from ODYSSEY (DTG) and IMPAACT2019 (3TC). Across all WBs, GM AUC_0–24 h_ and C_trough_ levels for DTG and GM AUC_0–24 h_ for 3TC were within the reference ranges from prior regulatory approvals for paediatric Tivicay® and Triumeq®. Most children had DTG C_trough_ levels above the EC90 target of 0.32 mg/L, comparable to ODYSSEY in similar WBs/doses and formulations.^21^^,^^22^ Three children had DTG C_trough_ level below this target, one in the 10–<14 kg WB and two in the ≥25 kg WB. All children exceeded the PA-IC90 of 0.064 mg/L, with the lowest recorded level at 0.25 mg/L. The observed variability in individual DTG levels is consistent with previous data in children, likely reflecting inter individual differences in metabolic enzyme activity. Although this study was not designed to assess covariates through modelling approaches, known factors such as body size and food intake were accounted for by applying WHO-recommended weight-band dosing and standardised fasting conditions based on dosing, respectively.

This study evaluated the adult 3TC dose (300 mg) in children weighing ≥20 kg, considering simplification and patient preference for a single tablet. This resulted in 72% higher 3TC GM AUC_0–24 h_ in the 20–<25 kg FCT WB compared to children on DTG/3TC dispersible tablets in the same WB in D3/Penta 21, and 61% higher than in IMPAACT 2019, both of which used a 3TC dose of 180 mg.^30^ However, the 3TC GM AUC_0–24 h_ in this WB was only 19% higher than in children weighing ≥25 kg in D3/Penta 21, receiving one adult DTG/3TC FCT, and 8% higher than in children in IMPAACT 2019, receiving adult DTG/ABC/3TC. While children receiving one adult DTG/3TC tablet in 20–<25 kg may be temporarily exposed to higher levels of 3TC, this increased exposure is transient due to rapid growth at this age. Importantly, no 3TC-related adverse events or clinically significant laboratory abnormalities were observed in this group. Although earlier studies indicated a potential link between 3TC concentrations and neutropenia in adults receiving high doses (20 mg/kg/day),^34^ no excess haematological toxicities were noted in children in the 20–<25 kg FCT WB compared to other WBs.

Both DTG/3TC fixed-dose combination formulations were well tolerated, with reassuring safety outcomes over 48 weeks. The findings align with paediatric ODYSSEY and IMPAACT 2019 trials.^30^^,^^35^ Notably, GEMINI trials of ART-naïve adults reported fewer drug-related adverse events and more favourable changes in renal and bone biomarkers amongst those receiving DTG + 3TC compared to triple drug regimen (DTG + TDF/FTC).^8^ However, some adult switch trials (e.g. TANGO, SALSA) showed a higher rate of AEs in the first 24–48 weeks post-switch to DTG and 3TC compared to continuing triple-drug regimens, which could be expected with a switch to a new regimen. Notably, this was a single arm substudy which aimed to assess PK and safety. Comparative safety outcomes, including laboratory parameters, will be evaluated by randomised arm in the main trial, which is expected to provide more robust data relative to currently used DTG-based three-drug regimens.^20^

A potential concern with the DTG/3TC 2DR regimen is the risk of treatment-emergent resistance, especially in populations with pre-existing mutations and those at risk for non-adherence, such as adolescents. However, recent real-world data and clinical trials in adults have shown no new INSTI resistance in individuals who are ART-naïve and minimal resistance in those with prior ART experience.^36^ Even in individuals with M184V/I mutations, virological failure rates remained low, suggesting that a 2DR regimen with DTG/3TC carries no increased risk of resistance compared to the standard 3DR regimens.^37^

A limitation of this study was the absence of pharmacokinetic curves for children in the lowest weight band (6–<10 kg WB), as no virologically suppressed children aged ≥2 years and weighing <10 kg were recruited to the main trial. This weight band was the lowest WHO dosing weight band that accommodated 2-year-olds on the lower percentiles for weight-for-age. We anticipated a low number of eligible participants, given the longer time infants take to achieve virological suppression. Despite the absence of data from the 6 to <10 kg weight band, the study's large sample size and diverse age and weight range strengthen the robustness of our findings. Interindividual variability in DTG and 3TC exposures was consistent with previous studies (ODYSSEY, IMPAACT2019), supporting the applicability of the WHO weight-band dosing approach of the majority of children.

Five major protocol deviations were identified in four children. In the two cases where deviations could have influenced the results, repeat intensive PK sampling was performed; thus, these deviations are not expected to have impacted the results. Additionally, four PK timepoints lacked concentration data for three children (two in 14–<20 kg WB and one in 20–<25 kg FCT WB), representing less than 1% of all samples and unlikely to affect overall findings.

In conclusion, the D3/Penta 21 pharmacokinetic sub-study demonstrated adequate DTG and 3TC drug exposures with reassuring safety profiles for WB-based dosing of DTG/3TC, supporting the licencing applications for dispersible and film-coated DTG/3TC formulations for paediatric use.

## Contributors

LB conceived the manuscript, and both LB and GT contributed to its draughting. LB primarily wrote the pharmacokinetic sections, while GT focused on safety. All other authors (MK, EK, GA, ID, TA, EV, AV, IW, DB, AN, EM, PA, DR, AK, CK, PM, MA, JB, MT, SW, CG, AC, DM, AB, MC, TJ, AT) made crucial revisions to and approved the final manuscript. Data were analysed and verified by study authors: pharmacokinetic data by LB; and safety data by GT. The senior authors (AC, DB, MC, TJ, AT) had full access to all data in the study and final responsibility for the decision to submit for publication.

## Data sharing statement

The D3/Penta 21 data are held at MRC CTU at UCL, which encourages optimal use of data by employing a controlled access approach to data sharing, incorporating a transparent and robust system to review requests and provide secure data access consistent with the relevant ethics committee approvals. All requests for data are considered and can be initiated by contacting mrcctu.ctuenquiries@ucl.ac.uk.

## Declaration of interests

Ann M. Buchanan and Justine Boles are employees of ViiV Healthcare and receives stock options from GSK. Isabelle Deprez is an employee of Certara, owns Certara shares, and is also a complementary worker for GSK. Anna Turkova and Man K. Chan receives core support from the UK Medical Research Council (grants MC_UU_00004/03). Avy Violari received funding for the parent study (institutional payments). Angela Colbers received grants from Gilead, ViiV, and Merck (via Radboudumc), and consulting fees from Gilead. David Burger received funding from ViiV for the PANNA study and a ViiV-sponsored lecture (both paid to institution). Moherndran Archary holds an NIHR Global Research Professorship. Justine Boles holds GSK shares. Pauline Amuge reports study support from PENTA Fondazione at Baylor-Uganda. Saskia de Wildt received institutional funding from EU IMI2, Roche, and the Gates Foundation. She is co-inventor on a pending patent (institution to receive any proceeds) and serves on advisory boards and in leadership roles for various academic and nonprofit organisations, mostly unpaid or institutionally compensated.

## Appendix A Supplementary data

Additional File 1 D3 Trial Team

Supplementary Material (pg no: 30–46)
