# Supplementary material for: Once-daily dolutegravir/lamivudine fixed-dose formulations in children living with HIV: a pharmacokinetic and safety sub-study nested in the open-label, multicentre, randomised, non-inferiority D3/PENTA 21 trial
Source: eBioMedicine. 2025 Sep 26;120:105929. doi: 10.1016/j.ebiom.2025.105929 (PMC12509724; doi:10.1016/j.ebiom.2025.105929)
Supplement: Additional File 1 D3 Trial Team [file mmc1.docx]

# D3 Trial Team

**Fondazione Penta ETS, Padova, Italy:** Carlo Giaquinto, Alessandra Nardone, Gabija Morkunaite

**University of Padova, Department of Women and Child Health, Padova, Italy:** Carlo Giaquinto

**MRC CTU at UCL:** Anna Turkova, Debbie Ford, Man Chan, Gabriela Toledo, Elizabeth James, Mags Thomason, Nazia Parkar, Iona White, Anna Parker, Anas Omar, Zainab Alkurwi, Moira Spyer, Katja Doerholt, Stephen Townsend, Hannah Sweeney, Margaret Hook, Alvin Daramola-Rose, Lee Barker, Lu Gao, Matteo Quartagno, Diana Gibb

**AMS – PHPT Research Collaboration, Faculty of Associated Medical Sciences, Chiang Mai University, Chiang Mai, Thailand:** Tim R. Cressey, Suwalai Chalermpantmetagul, Rukchanok Peongjakta, Ketwarin Pongkawong, Worathip Sripaoraya, Warunee Khamjakkaew, Namthip Kruenual, Pra-ornsuda Sukrakanchana, Ampika Kaewbundit

**Baylor College of Medicine Children’s Foundation, Uganda:** Adeodata R. Kekitiinwa, Pauline Amuge, Christine Namugwanya, Lameck Kiyimba, Resty Babirye Okello, Florence Namuli, Ronald Nabimba, Angella Baita, Dickson Bbuye, Susan Tukamuhebwa, Rachael Namuddu Kikabi, Rose Jacqueline Kadhuba, Henry Balwa, Sarah Nabukalu, Muzamil Nsibuka Kisekka, Anthony Kirabira, Lekku Lawrence, Judith Tikabibamu, Maria Benita Aino, Gerald Agaba Muzorah, Collins Mujyanama, Annet Nalugo, Priscilla Namubiru

**Joint Clinical Research Centre, Uganda:** Cissy Kityo, Victor Musiime, Elizabeth Kaudha, Annet Nanduudu, Henry Mugerwa, Caroline Otike, Emmanuel Mujyambere, Dridah Nakiboneka, Barbara Mukanza, Julius Tumusiime, Onen Gilbert, Ritah Mbabazi, Abigail Atwine, Priscilla Kyobutungi, Juliet Ategeka, Alex Musiime, Sharif Musumba, Rashidah Nazzinda, Nicholas Wangwe, Phyllis Mwesigwa, Diana Rutebarika, Jameena Elsauko, Mangadalen Nansaigi, Mercy Tukamushaba, Alice Mulindwa, Aidah Nakalyango, Ocitti Paul, Christine Nambi, Milly Ndigendawani, Mariam Naabalamba, Eram David, Odochi Denis, Baliruno David, Ezra Lutalo, Eddie Rubanga, Josephine Namusanje, Josephine Kobusingye, Disan Mulima, Maria Nannungi, Faith Balmoi, Charles Draleku, Faith Mbasani, Crispus Katemba, Juliet Ankunda, Julian Tusiime, Benson Ouma Juma

**Makerere University – John Hopkins University Research Collaboration, Uganda:** Philippa Musoke, Grace Miriam Ahimbisibwe, Hajira Kataike, Winnie Nansamba, David Balamusani, Rosemary Namwanje, Enoch Mulwanyi, Gerald Bright Businge, Maxensia Owor, Aziida Nabukeera, Ruth Nakku, Zainab Nakivumbi Nassoma, Immaculate Nayiga Serunjogi, Barbara Musoke Nakirya, Sarah Nakabuye, Erinah Kyomukama, Rebecca Wampamba, Stella Nalusiba, Emmanuel Mayanja, Donald Wagaana, Zaam Zinda Nakawungu, Sarah Babirye Ssebabi, Olivia Higiro Kaboggoza, Edith Nabawubye, Mildred Kabasonga, Harriet Namusisi, Judith Nampewo, Agnes Mary Mugagga, Richard Isabirye, Francis Sserugo, Annet Kawuma, Agnes Namuddu, Joanita Nankya Baddokwaya, Juliet Nanyonjo, Winifred Kaahwa, Maria Musisi, Paula Mubiru Namayanja, Doreen Twenatwine, Robert Byuma, Winifred Luwedde, Margaret Mugenyi, Joseph Mutebo, Francis Katongole, Fabian Okello, Max Kiwewa, Ronald Okwera, Derick Balungi, Obed Tumwizere, Teopista Nakyanzi, Ann Kankindi, Maria Janine Nambusi, Ivan Rukundo, Henry Odyek, Barnabas Weere, Bosco Kafufu, David Ssebunya, Africano Kamugisha, Emmanual Hakizimana, Charles Nyende, Evelyn Akurut, Johnson Tumwesigye, Brenda Catherine Kakayi, Rebecca Sakwa, Mark Ssenyonga, Joseph Semakula, Joyce Mwebaza, Judith Kainza, Miscah Babirye Otim

**Durban International Clinical Research Site, Enhancing Care Foundation, South Africa:** Moherndran Archary, Rosie Mngqibisa, Tiyara Arumugam, Sundrapragasen Pillay, Raziya Bobat, Nombuso Nkosi, Nozibusiso Rejoice Mosia, Sheleika Singh, Shingirai Chimene, Jabu Mkhulise, Innocentia Thandokuhle Mncube, Zethu Mnyandu, Michelle Moodley, Popi Shabalala, Simangele Bengu, Ida Elizabeth Mundhree

**Perinatal HIV Research Unit, Matlosana, South Africa:** Ebrahim Variava, Tumelo Moloantoa, Nadia Sabet, Ryan Sabet, Modiehi Mosala, Itumeleng Holele, Eva Mogotsi, Angelinah Montwedi, Serame Mokoena, Keabetswe Kotsokoane, Dineo Rampai, Avy Violari, Lerato Maretlwa, Palesa Tshipunyane, Iris Matotong, Abraham Mammwn Pattamukkil, Nkazimulo Xulu, Sthembiso Mhlanga, Ofhani Harmious Makhari, Gifty Okyere Manu, Zukisa Mpeluza, Tumelo Moloantoa, Mbusiseni Ngema, Abdul Kaka, Rieta Stokes, Nadia Bellingan, Linique Le Grange, Leoni Stytler, Zakkiyya Jeeva

**Perinatal HIV Research Unit, Soweto, South Africa:** Avy Violari, Afaaf Liberty, Mandisa Nyati, Haseena Cassim, Sisinyana Ruth Mathiba, Lindiwe Maseko, Precious Ndebele, Jackie Brown, Emily Lebotsa, Deirdre Josipovic, Mantwa Kunene, Tryphinah Madonsela, Nasreen Abrahams, Zaakirah Essack, Zandisile Mtshali, Dipuo Dhlomoza, Thabile Degracia Hlomuka, Valerie Khemese

**Chiangrai Prachanukroh Hospital, Thailand:** Pradthana Ounchanum, Suchada Ruenglerdpong, Areerat Kongponoi, Kanyanee Kaewmamueng, Warunee Srisuk, Yupawan Thaweesombat, Sukanda Denjanta, Jutarat Thewsoongnoen, Naowarat Kunyanone

**Kalasin Hospital, Thailand:** Sakulrat Srirojana, Doungjai Donngern, Petcharat Phunkhum, Arisara Kamkoonmongkol, Thananya Naksomboon, Nonthaporn Na Kalasin

**Khon Kaen Hospital, Thailand:** Ussanee Srirompotong, Athiporn Rungsapphaiboon, Wallapa Daechasatain, Wanchalerm Boonsub, Patamawadee Sudsaard, Thunyasiri Dechboran, Manthana Mitchai, Thanawat Samranphit, Orapin Wannasri, Kriangkrai Kongsuk

**Nakornping Hospital, Thailand:** Suparat Kanjanavanit, Rujirek Kamolrattana, Thannapat Chankun, Jiraporn Punyayen, Chayakorn Saewtrakool, Pacharaporn Yingyong, Raungwit Junkaew, Benjawan Thomyota

**Prapokklao Hospital, Thailand:** Kanokkorn Sawasdichai, Chaiwat Ngampiyaskul, Nantika Paiboon, Benjama I-nala, Wanna Chamjamrat, Pisut Greetanukroh, Chanthaporn Imbumroong, Sasipass Khannak, Rattana Chahmeanprabnakorn

**Birmingham Heartlands Hospital, UK:** Steven Welch, Ezgi Seager, Yvonne Beuvink, Melanie Rooney, Laura Thrasyvoulou, Katie Price, George Gavin, Sue Fagg, Baldip Kaur, Molly Williams,

**Great Ormond Street Hospital, UK:** Anna Turkova, Alasdair Bamford, Delane Shingadia, Jade Sugars, Olamide Alimi, Carolyn Chan, Kelly Cripps, Sabina Zahed, John Koroma

**Hospital Sant Joan de Déu, Spain:** Clàudia Fortuny, Antoni Noguera-Julian, Anna Vazquez Marchan, Cristina López Hidalgo, Sílvia Cuadras Ferrando, Alba Murciano, Miriam Coto, Kenia Sánchez, Bonaventura Ruiz

**Hospital Universitario 12 de Octubre, Spain:** Pablo Rojo, Cristina Epalza, Luis Prieto, Jose Tomas Ramos, Ianire Gallego, Verónica Martín, Lilit Manukyan, Manuel Gijon

**PK substudies:** David Burger, Angela Colbers, Tom Jacobs, Lisanne Bevers

**Pharmacogenomics substudy:** Dan Carr, Andrew Owen, Rebecca Jensen

**Virology substudy:** Eleni Nastouli, Moira Spyer, Matt Byott, Ian Botha

**Health Economics substudy:** Paul Revill, Simon Walker

**Youth Trials Board:** Magda Conway, Lungile Jafta, Mercy Shibemba

**Trial Steering Committee Members:** Hermione Lyall*,* Elizabeth Maleche Obimbo*,* Theodore Ruel*,* Alex Compagnucci*,* Imelda Mahaka, Fanele Bulose*,* Gugulethu Bomela, Anna Turkova, Cissy Kityo, Tim Cressey*,* Avy Violari

**Data Monitoring Committee Members:** Anton Pozniak, Jane Crawley, Rodolphe Thiébaut, Helen McIlleron (for consideration of PK substudy)
