# Supplementary material for: Once-daily dolutegravir/lamivudine fixed-dose formulations in children living with HIV: a pharmacokinetic and safety sub-study nested in the open-label, multicentre, randomised, non-inferiority D3/PENTA 21 trial
Source: eBioMedicine. 2025 Sep 26;120:105929. doi: 10.1016/j.ebiom.2025.105929 (PMC12509724; doi:10.1016/j.ebiom.2025.105929)

**Supplementary materials**

Table S1. Baseline characteristics by enrolment weight band (safety population)

| **Number of participants** | **Overall** | **10-<14 kg**  (20/120 mg DT) | **14-<20 kg**  (25/150 mg DT) | **20-<25 kg DT**  (30/180 mg DT) | **20-<25 kg FCT**  (50/300 mg FCT) | **25-<40 kg**  (50/300 mg FCT) |
| --- | --- | --- | --- | --- | --- | --- |
|  | **N=82** | **n=12** | **n=22** | **n=12** | **n=14** | **n=22** |
| Sex |  |  |  |  |  |  |
| Male | 34 (41%) | 6 (50%) | 9 (41%) | 3 (25%) | 7 (50%) | 9 (41%) |
| Female | 48 (59%) | 6 (50%) | 13 (59%) | 9 (75%) | 7 (50%) | 13 (59%) |
| Race (black) | 82 (100%) | 12 (100%) | 22 (100%) | 12 (100%) | 14 (100%) | 22 (100%) |
| Age (years) |  |  |  |  |  |  |
| Median (IQR) | 7.1 (5.0, 10.4) | 3.3 (2.6, 3.7) | 5.4 (4.7, 6.5) | 7.4 (7.1, 8.3) | 8.1 (7.1, 9.1) | 12.2 (10.7, 13.3) |
| Range | [2.3-13.8] | [2.3-4.5] | [3.7-7.2] | [6.7-11.4] | [5.5-11.1] | [7.2-13.8] |
| Weight (kg) |  |  |  |  |  |  |
| Median (IQR) | 21.4 (18.0, 26.9) | 12.9 (12.6, 13.2) | 18.4 (16.8, 19.3) | 22.1 (21.1, 22.5) | 22.6 (21.5, 23.3) | 31.6 (29.1, 34.9) |
| Range | [12.0-38.5] | [12.0-13.6] | [14.2-19.8] | [20.5-22.9] | [20.0-24.9] | [26.5-38.5] |
| Weight-for-age § | 61 | 12 | 22 | 11 | 13 | 3 |
| Median (IQR) | -0.7 (-1.0, -0.2) | -1.0 (-1.5, -0.3) | -0.6 (-0.8, -0.3) | -0.5 (-1.1, 0.0) | -0.8 (-1.2, -0.1) | 0.4 (-0.3, 1.0) |
| Range | [-2.9-1.0] | [-2.2--0.1] | [-1.4-0.4] | [-2.9-0.2] | [-2.3-0.3] | [-0.3-1.0] |
| -3 to less than -2 | 3 (4%) | 1 (8%) | 0 (0%) | 1 (8%) | 1 (7%) | 0 (0%) |
| -2 to less than 0 | 48 (59%) | 11 (92%) | 18 (82%) | 7 (58%) | 11 (79%) | 1 (5%) |
| 0 or more | 10 (12%) | 0 (0%) | 4 (18%) | 3 (25%) | 1 (7%) | 2 (9%) |
| Missing | 21 (26%) | 0 (0%) | 0 (0%) | 1 (8%) | 1 (7%) | 19 (86%) |
| Height (cm) |  |  |  |  |  |  |
| Median (IQR) | 118 (107, 131) | 90 (88, 94) | 109 (103, 116) | 119 (118, 122) | 118 (116, 126) | 141 (134, 147) |
| Range | [84-153] | [84-101] | [98-125] | [116-123] | [110-130] | [131-153] |
| Height-for-age§ |  |  |  |  |  |  |
| Median (IQR) | -1.0 (-1.7, -0.3) | -1.2 (-1.7, -1.0) | -0.4 (-1.5, 0.1) | -0.9 (-1.1, -0.5) | -1.0 (-1.8, -0.3) | -1.2 (-1.8, -0.1) |
| Range | [-4.2-3.6] | [-3.7--0.5] | [-2.5-3.6] | [-4.0--0.4] | [-4.2-0.4] | [-2.9-2.2] |
| Less than <-3 | 4 (5%) | 2 (17%) | 0 (0%) | 1 (8%) | 1 (7%) | 0 (0%) |
| -3 to less than -2 | 9 (11%) | 0 (0%) | 2 (9%) | 1 (8%) | 2 (14%) | 4 (18%) |
| -2 to less than 0 | 58 (71%) | 10 (83%) | 14 (64%) | 10 (83%) | 10 (71%) | 14 (64%) |
| 0 or more | 11 (13%) | 0 (0%) | 6 (27%) | 0 (0%) | 1 (7%) | 4 (18%) |
| BMI (kg/m2) |  |  |  |  |  |  |
| Median (IQR) | 15.6 (14.6, 16.6) | 15.7 (14.6, 16.6) | 14.7 (14.1, 15.7) | 15.1 (14.7, 16.3) | 15.8 (14.4, 16.8) | 16.2 (15.1, 17.3) |
| Range | [11.9-19.3] | [12.4-18.8] | [11.9-19.3] | [13.8-17.0] | [13.2-18.1] | [12.9-17.5] |
| BMI-for-age § |  |  |  |  |  |  |
| Median (IQR) | -0.4 (-0.9, 0.3) | 0.1 (-0.7, 0.8) | -0.5 (-0.9, 0.2) | -0.4 (-0.6, 0.5) | -0.1 (-0.9, 0.7) | -0.8 (-1.2, -0.4) |
| Range | [-3.9-2.3] | [-2.3-2.3] | [-3.1-2.2] | [-1.7-0.9] | [-2.5-1.4] | [-3.9-0.6] |
| Less than <-3 | 2 (2%) | 0 (0%) | 1 (5%) | 0 (0%) | 0 (0%) | 1 (5%) |
| -3 to less than -2 | 3 (4%) | 1 (8%) | 1 (5%) | 0 (0%) | 1 (7%) | 0 (0%) |
| -2 to less than 0 | 47 (57%) | 4 (33%) | 11 (50%) | 8 (67%) | 7 (50%) | 17 (77%) |
| 0 or more | 30 (37%) | 7 (58%) | 9 (41%) | 4 (33%) | 6 (43%) | 4 (18%) |
| DTG exposure prior to D3/Penta 21 entry |  |  |  |  |  |  |
| <1 month or no use | 9 (11%) | 2 (17%) | 4 (18%) | 0 (0%) | 3 (21%) | 0 (0%) |
| >=1 month | 73 (89%) | 10 (83%) | 18 (82%) | 12 (100%) | 11 (79%) | 22 (100%) |
| Country |  |  |  |  |  |  |
| South Africa | 12 (15%) | 3 (25%) | 3 (14%) | 0 (0%) | 6 (43%) | 0 (0%) |
| Uganda | 70 (85%) | 9 (75%) | 19 (86%) | 12 (100%) | 8 (57%) | 22 (100%) |

§ WHO Child Growth Charts and WHO Reference 2007 Charts. Weight for age calculated for those younger than 10 years and BMI for age calculated for those younger than 19 years. If children were 10 years or older, weight for age will be missing

Table S2. Listing of intensive pharmacokinetic protocol deviations in the 72 participants included in the PK analysis

| Weight band | Dose/  formulation | Grade | Deviation | Reason for deviation | Pharmacokinetic curves |
| --- | --- | --- | --- | --- | --- |
| 14 - <20 kg | 25/150 mg  DT | Major | Intensive PK deviation - blood sample(s) not taken at the required time points or sample(s) clotted/not stored | Other |  |
| 14 - <20 kg | 25/150 mg  DT | Major | Intensive PK deviation - blood sample(s) not taken at the required time points or sample(s) clotted/not stored | Other |  |
| 14 - <20 kg | 25/150 mg  DT | Major | Intensive PK deviation - Dispersible DTG/3TC tablet not dispersed correctly as per guidance in Intensive 24H PK and PD Study of DTG/3TC MOP on day of Intensive PK visit. | Investigator error | Intensive PK sampling was repeated, and pharmacokinetic curves from the repeat visit contributed to the analysis |
| 14 - <20 kg | 25/150 mg  DT | Major | Intensive PK deviation - participant has not taken the weight-appropriate dose of DTG/3TC once daily in the morning or the three days prior to the Intensive PK day, and Intensive PK visit still carried out | Investigator error | Intensive PK sampling was repeated, and pharmacokinetic curves from the repeat visit contributed to the analysis |
| 20 - <25 kg | 50/300 mg  FCT | Major | Intensive PK deviation - blood sample(s) not taken at the required time points or sample(s) clotted/not stored | Laboratory error |  |

Note: there were no critical PDs reported in the 72 participants included in the PK analysis

Table S3. Listing of serious adverse events and grade ≥3 clinical adverse events reported over the safety reporting period

| Weight band | Dose/  formulation | Treatment duration (weeks) at onset | Serious adverse event | MedDRA System organ class | MedDRA Preferred term | Maximum grade | ART-modifying | Relatedness to ART |
| --- | --- | --- | --- | --- | --- | --- | --- | --- |
| 14-<20kg | 25/150 mg DT | 4.0 | Yes | Infections and infestations | Cerebral malaria | Grade 4 | No | Not related |
| 20-<25kg † | 50/300 mg FCT | 30.9 | Yes | Infections and infestations | Pneumonia | Grade 3 | No | Not related |
| 20-<25kg | 50/300 mg FCT | 13.7 | Yes | Respiratory, thoracic and mediastinal disorders | Adenoidal hypertrophy | Grade 2 | No | Not related |
| 20-<25kg † | 50/300 mg FCT | 31.1 | Yes | Respiratory, thoracic and mediastinal disorders | Cor pulmonale | Grade 3 | No | Not related |
| 14-<20kg | 25/150 mg DT | 10.3 | No | Gastrointestinal disorders | Diarrhoea | Grade 3 | No | Not related |
| 20-<25kg | 30/180 mg DT | 51.1 | No | Blood and lymphatic system disorders | Febrile neutropenia | Grade 3 | No | Not related |
| ≥25kg | 50/300 mg FCT | 34.9 | No | Blood and lymphatic system disorders | Sickle cell anaemia with crisis | Grade 3 | No | Not related |

† One child experienced two SAEs (pneumonia and cor pulmonale) in the 20-<25 kg WB (50/300 mg FCT).

Table S4. Listing of grade ≥3 treatment emergent laboratory anomalies reported over the safety reporting period

| Weight band at time of event | Dose/formulation | Analyte | Grade | Treatment duration (weeks) at time of event |
| --- | --- | --- | --- | --- |
| 10-<14kg | 20/120 mg DT | Neutrophils | 3 | 12 |
| 14-<20kg† | 25/150 mg DT | Creatinine clearance | 3 | 4 |
| 14-<20kg | 25/150 mg DT | Haemoglobin | 3 | 24.1 |
| 14-<20kg | 25/150 mg DT | Neutrophils | 3 | 48.3 |
| 20-<25kg† | 30/180 mg DT | Neutrophils | 3 | 51.6 |

† Two laboratory events (creatinine clearance and neutrophils) occurred in the same participant in the 14-<20 kg and 20-<25 kg WB, respectively.

Table S5. Summary of adverse events of grade ≥1 by MedDRA system organ class (SOC) and preferred term (PT) over the safety reporting period by current weight band and formulation at time of the event (highest grade in each participant by current weight band/formulation and SOC/PT)

| **System organ class/Preferred term** | **Grade 1** | **Grade 2** | **Grade 3** | **Grade 4** | **Grade 5** | **Total** |
| --- | --- | --- | --- | --- | --- | --- |
| **10-<14 kg (n=12)**  (20/120 mg DT) |  |  |  |  |  |  |
| **Total number of participants with any event** | **4** | **0** | **0** | **0** | **0** | **4** |
| **Infections and infestations** | **4** | **0** | **0** | **0** | **0** | **4** |
| Gastroenteritis | 1 | 0 | 0 | 0 | 0 | 1 |
| Nasopharyngitis | 1 | 0 | 0 | 0 | 0 | 1 |
| Upper respiratory tract infection | 2 | 0 | 0 | 0 | 0 | 2 |
| **14-<20 kg (n=33)**  (25/150 mg DT) |  |  |  |  |  |  |
| **Total number of participants with any event** | **10** | **8** | **1** | **1** | **0** | **20** |
| **Infections and infestations** | **8** | **7** | **0** | **1** | **0** | **16** |
| Cerebral malaria | 0 | 0 | 0 | 1 | 0 | 1 |
| Gastroenteritis | 1 | 0 | 0 | 0 | 0 | 1 |
| Nasopharyngitis | 1 | 0 | 0 | 0 | 0 | 1 |
| Pharyngitis | 1 | 0 | 0 | 0 | 0 | 1 |
| Tinea capitis | 0 | 1 | 0 | 0 | 0 | 1 |
| Tonsillitis | 0 | 2 | 0 | 0 | 0 | 2 |
| Upper respiratory tract infection | 7 | 5 | 0 | 0 | 0 | 12 |
| Urinary tract infection | 1 | 0 | 0 | 0 | 0 | 1 |
| Varicella | 2 | 0 | 0 | 0 | 0 | 2 |
| Viral infection | 2 | 0 | 0 | 0 | 0 | 2 |
| **Skin and subcutaneous tissue disorders** | **2** | **1** | **0** | **0** | **0** | **3** |
| Dermatitis contact | 1 | 0 | 0 | 0 | 0 | 1 |
| Pruritus | 1 | 0 | 0 | 0 | 0 | 1 |
| Rash vesicular | 0 | 1 | 0 | 0 | 0 | 1 |
| **Gastrointestinal disorders** | **1** | **1** | **1** | **0** | **0** | **3** |
| Dental caries | 1 | 0 | 0 | 0 | 0 | 1 |
| Diarrhoea | 0 | 0 | 1 | 0 | 0 | 1 |
| Stomatitis | 0 | 1 | 0 | 0 | 0 | 1 |
| Toothache | 1 | 0 | 0 | 0 | 0 | 1 |
| Vomiting | 1 | 0 | 0 | 0 | 0 | 1 |
| **Respiratory, thoracic and mediastinal disorders** | **1** | **0** | **0** | **0** | **0** | **1** |
| Cough | 1 | 0 | 0 | 0 | 0 | 1 |
| **Eye disorders** | **1** | **0** | **0** | **0** | **0** | **1** |
| Conjunctivitis allergic | 1 | 0 | 0 | 0 | 0 | 1 |
| **General disorders and administration site conditions** | **1** | **0** | **0** | **0** | **0** | **1** |
| Decreased appetite | 1 | 0 | 0 | 0 | 0 | 1 |
| **Nervous system disorders** | **1** | **0** | **0** | **0** | **0** | **1** |
| Headache | 1 | 0 | 0 | 0 | 0 | 1 |
| **20-<25 kg DT (n=17)**  (30/180 mg DT) |  |  |  |  |  |  |
| **Total number of participants with any event** | **7** | **3** | **1** | **0** | **0** | **11** |
| **Infections and infestations** | **5** | **3** | **0** | **0** | **0** | **8** |
| Folliculitis | 1 | 0 | 0 | 0 | 0 | 1 |
| Mumps | 1 | 0 | 0 | 0 | 0 | 1 |
| Nasopharyngitis | 1 | 0 | 0 | 0 | 0 | 1 |
| Pharyngitis | 1 | 0 | 0 | 0 | 0 | 1 |
| Upper respiratory tract infection | 2 | 2 | 0 | 0 | 0 | 4 |
| Urinary tract infection | 0 | 1 | 0 | 0 | 0 | 1 |
| **Skin and subcutaneous tissue disorders** | **1** | **1** | **0** | **0** | **0** | **2** |
| Rash | 0 | 1 | 0 | 0 | 0 | 1 |
| Urticaria | 1 | 0 | 0 | 0 | 0 | 1 |
| **Gastrointestinal disorders** | **1** | **1** | **0** | **0** | **0** | **2** |
| Abdominal pain | 1 | 0 | 0 | 0 | 0 | 1 |
| Diarrhoea | 0 | 1 | 0 | 0 | 0 | 1 |
| **Injury, poisoning and procedural complications** | **0** | **2** | **0** | **0** | **0** | **2** |
| Contusion | 0 | 1 | 0 | 0 | 0 | 1 |
| Muscle strain | 0 | 1 | 0 | 0 | 0 | 1 |
| **Blood and lymphatic system disorders** | **0** | **0** | **1** | **0** | **0** | **1** |
| Febrile neutropenia | 0 | 0 | 1 | 0 | 0 | 1 |
| **Musculoskeletal and connective tissue disorders** | **1** | **0** | **0** | **0** | **0** | **1** |
| Neck pain | 1 | 0 | 0 | 0 | 0 | 1 |
| **20-<25 kg FCT (n=22)**  (50/300 mg FCT) |  |  |  |  |  |  |
| **Total number of participants with any event** | **4** | **3** | **1** | **0** | **0** | **8** |
| **Infections and infestations** | **3** | **2** | **1** | **0** | **0** | **6** |
| Lower respiratory tract infection | 1 | 2 | 0 | 0 | 0 | 3 |
| Nasopharyngitis | 1 | 0 | 0 | 0 | 0 | 1 |
| Pneumonia | 0 | 0 | 1 | 0 | 0 | 1 |
| Respiratory tract infection viral | 1 | 0 | 0 | 0 | 0 | 1 |
| Upper respiratory tract infection | 1 | 1 | 0 | 0 | 0 | 2 |
| **Skin and subcutaneous tissue disorders** | **3** | **0** | **0** | **0** | **0** | **3** |
| Dermatitis atopic | 1 | 0 | 0 | 0 | 0 | 1 |
| Papular pruritic eruption of HIV | 1 | 0 | 0 | 0 | 0 | 1 |
| Rash | 1 | 0 | 0 | 0 | 0 | 1 |
| **Respiratory, thoracic and mediastinal disorders** | **0** | **1** | **1** | **0** | **0** | **2** |
| Adenoidal hypertrophy | 0 | 1 | 0 | 0 | 0 | 1 |
| Cor pulmonale | 0 | 0 | 1 | 0 | 0 | 1 |
| **25-<40 kg (n=32)**  (50/300 mg FCT) |  |  |  |  |  |  |
| **Total number of participants with any event** | **11** | **3** | **1** | **0** | **0** | **15** |
| **Infections and infestations** | **10** | **3** | **0** | **0** | **0** | **13** |
| Body tinea | 1 | 0 | 0 | 0 | 0 | 1 |
| Gastroenteritis | 1 | 0 | 0 | 0 | 0 | 1 |
| Malaria | 0 | 1 | 0 | 0 | 0 | 1 |
| Papular pruritic eruption of HIV | 0 | 1 | 0 | 0 | 0 | 1 |
| Paronychia | 1 | 0 | 0 | 0 | 0 | 1 |
| Plasmodium falciparum infection | 1 | 0 | 0 | 0 | 0 | 1 |
| Tinea capitis | 1 | 0 | 0 | 0 | 0 | 1 |
| Upper respiratory tract infection | 6 | 2 | 0 | 0 | 0 | 8 |
| Urinary tract infection | 1 | 0 | 0 | 0 | 0 | 1 |
| Varicella | 1 | 0 | 0 | 0 | 0 | 1 |
| Vulvovaginal candidiasis | 1 | 0 | 0 | 0 | 0 | 1 |
| **Skin and subcutaneous tissue disorders** | **1** | **0** | **0** | **0** | **0** | **1** |
| Dermatitis | 1 | 0 | 0 | 0 | 0 | 1 |
| **Gastrointestinal disorders** | **1** | **0** | **0** | **0** | **0** | **1** |
| Abdominal pain | 1 | 0 | 0 | 0 | 0 | 1 |
| **Respiratory, thoracic and mediastinal disorders** | **1** | **0** | **0** | **0** | **0** | **1** |
| Productive cough | 1 | 0 | 0 | 0 | 0 | 1 |
| **Injury, poisoning and procedural complications** | **1** | **0** | **0** | **0** | **0** | **1** |
| Soft tissue injury | 1 | 0 | 0 | 0 | 0 | 1 |
| **Blood and lymphatic system disorders** | **0** | **0** | **1** | **0** | **0** | **1** |
| Sickle cell anaemia with crisis | 0 | 0 | 1 | 0 | 0 | 1 |
| **Musculoskeletal and connective tissue disorders** | **1** | **0** | **0** | **0** | **0** | **1** |
| Pain in extremity | 1 | 0 | 0 | 0 | 0 | 1 |

Results are presented by current weight band and formulation; hence, participants may appear in more than one weight band and formulation. This is primarily due to weight gain and transitioning into the next weight band over the safety reporting period. For example, a participant in 10-14Kg weight band gains weight over follow-up and moves to 14-20Kg. These are 2 current weight band/formulation periods for this participant. Events in each of these two current weight band/formulations are reported. The safety reporting period includes time from first fixed-dose combination DTG/3TC dose to the earliest of end of week 48 visit window or permanent DTG/3TC discontinuation. The highest clinical event grade by MedDRA SOC and PT is reported for each participant in current weight band and formulation at time of the event. For example, a participant in the 14-<20 kg weight band who has two events with the same PT (URTI) grade 1 and 2 will be counted as one grade 2 URTI in that weight band and formulation, as that is the clinical event with highest grade with the same PT.

Table S6. Summary of post-baseline emergent laboratory anomalies of grade ≥1 by current weight band and formulation over the safety reporting period (highest grade in each participant by current weight band/formulation for each analyte)

|  | **10-<14 kg (20/120 mg DT)** | **14-<20 kg (25/150 mg DT)** | **20-<25**  **(30/180 m**  **g DT)** | **20-<25**  **(50/300 mg FCT)** | **25-<40 kg**  **(50/300 mg FCT)** | **Total** |
| --- | --- | --- | --- | --- | --- | --- |
| **Albumin (N)** | **12** | **31** | **17** | **21** | **32** | **113** |
| Grade 1 | 0 | 2 (6%) | 0 | 0 | 2 (6%) | 4 (4%) |
| Grade 2 | 0 | 0 | 0 | 1 (5%) | 0 | 1 (1%) |
| Grade 3 | 0 | 0 | 0 | 0 | 0 | 0 |
| Grade 1 | 0 | 0 | 0 | 0 | 0 | 0 |
| Grade 1-4 (total) | 0 | 2 (6%) | 0 | 1 (5%) | 2 (6%) | 5 (4%) |
| **Alanine aminotransferase (N)** | **12** | **31** | **17** | **21** | **32** | **113** |
| Grade 1 | 1 (8%) | 4 (13%) | 0 | 0 | 0 | 5 (4%) |
| Grade 2 | 0 | 1 (3%) | 1 (6%) | 0 | 1 (3%) | 3 (3%) |
| Grade 3 | 0 | 0 | 0 | 0 | 0 | 0 |
| Grade 1 | 0 | 0 | 0 | 0 | 0 | 0 |
| Grade 1-4 (total) | 1 (8%) | 5 (16%) | 1 (6%) | 0 | 1 (3%) | 8 (7%) |
| **Aspartate aminotransferase (N)** | **12** | **31** | **17** | **21** | **32** | **113** |
| Grade 1 | 0 | 4 (13%) | 1 (6%) | 0 | 1 (3%) | 6 (5%) |
| Grade 2 | 0 | 1 (3%) | 0 | 0 | 0 | 1 (1%) |
| Grade 3 | 0 | 0 | 0 | 0 | 0 | 0 |
| Grade 1 | 0 | 0 | 0 | 0 | 0 | 0 |
| Grade 1-4 (total) | 0 | 5 (16%) | 1 (6%) | 0 | 1 (3%) | 7 (6%) |
| **Alkaline phosphatase (N)** | **12** | **31** | **17** | **21** | **32** | **113** |
| Grade 1 | 2 (17%) | 4 (13%) | 3 (18%) | 2 (10%) | 6 (19%) | 17 (15%) |
| Grade 2 | 0 | 0 | 0 | 0 | 1 (3%) | 1 (1%) |
| Grade 3 | 0 | 0 | 0 | 0 | 0 | 0 |
| Grade 1 | 0 | 0 | 0 | 0 | 0 | 0 |
| Grade 1-4 (total) | 2 (17%) | 4 (13%) | 3 (18%) | 2 (10%) | 7 (22%) | 18 (16%) |
| **Bilirubin (N)** | **12** | **31** | **17** | **21** | **32** | **113** |
| Grade 1 | 0 | 0 | 3 (18%) | 0 | 1 (3%) | 4 (4%) |
| Grade 2 | 0 | 0 | 0 | 0 | 0 | 0 |
| Grade 3 | 0 | 0 | 0 | 0 | 0 | 0 |
| Grade 1 | 0 | 0 | 0 | 0 | 0 | 0 |
| Grade 1-4 (total) | 0 | 0 | 3 (18%) | 0 | 1 (3%) | 4 (4%) |
| **Creatinine (N)** | **12** | **31** | **17** | **21** | **32** | **113** |
| Grade 1 | 0 | 3 (10%) | 2 (12%) | 2 (10%) | 1 (3%) | 8 (7%) |
| Grade 2 | 1 (8%) | 2 (6%) | 0 | 0 | 0 | 3 (3%) |
| Grade 3 | 0 | 0 | 0 | 0 | 0 | 0 |
| Grade 1 | 0 | 0 | 0 | 0 | 0 | 0 |
| Grade 1-4 (total) | 1 (8%) | 5 (16%) | 2 (12%) | 2 (10%) | 1 (3%) | 11 (10%) |
| **Creatinine clearance (N)** | **12** | **31** | **17** | **21** | **32** | **113** |
| Grade 1 | 0 | 0 | 0 | 0 | 0 | 0 |
| Grade 2 | 0 | 6 (19%) | 2 (12%) | 2 (10%) | 4 (12%) | 14 (12%) |
| Grade 3 | 0 | 1 (3%) | 0 | 0 | 0 | 1 (1%) |
| Grade 1 | 0 | 0 | 0 | 0 | 0 | 0 |
| Grade 1-4 (total) | 0 | 7 (23%) | 2 (12%) | 2 (10%) | 4 (12%) | 15 (13%) |
| **Cholesterol (N)** | **2** | **21** | **12** | **14** | **31** | **80** |
| Grade 1 | 0 | 1 (5%) | 0 | 0 | 2 (6%) | 3 (4%) |
| Grade 2 | 0 | 0 | 0 | 0 | 1 (3%) | 1 (1%) |
| Grade 3 | 0 | 0 | 0 | 0 | 0 | 0 |
| Grade 1 | 0 | 0 | 0 | 0 | 0 | 0 |
| Grade 1-4 (total) | 0 | 1 (5%) | 0 | 0 | 3 (10%) | 4 (5%) |
| **LDL (N)** | **2** | **21** | **12** | **14** | **31** | **80** |
| Grade 1 | 0 | 2 (10%) | 1 (8%) | 1 (7%) | 3 (10%) | 7 (9%) |
| Grade 2 | 0 | 0 | 0 | 1 (7%) | 0 | 1 (1%) |
| Grade 3 | 0 | 0 | 0 | 0 | 0 | 0 |
| Grade 1 | 0 | 0 | 0 | 0 | 0 | 0 |
| Grade 1-4 (total) | 0 | 2 (10%) | 1 (8%) | 2 (14%) | 3 (10%) | 8 (10%) |
| **Triglycerides (N)** | **2** | **21** | **12** | **14** | **31** | **80** |
| Grade 1 | 1 (50%) | 3 (14%) | 1 (8%) | 0 | 3 (10%) | 8 (10%) |
| Grade 2 | 0 | 2 (10%) | 0 | 0 | 0 | 2 (2%) |
| Grade 3 | 0 | 0 | 0 | 0 | 0 | 0 |
| Grade 1 | 0 | 0 | 0 | 0 | 0 | 0 |
| Grade 1-4 (total) | 1 (50%) | 5 (24%) | 1 (8%) | 0 | 3 (10%) | 10 (12%) |
| **Lactate (N)** | **12** | **28** | **15** | **17** | **25** | **97** |
| Grade 1 | 3 (25%) | 5 (18%) | 7 (47%) | 6 (35%) | 4 (16%) | 25 (26%) |
| Grade 2 | 0 | 1 (4%) | 0 | 0 | 0 | 1 (1%) |
| Grade 3 | 0 | 0 | 0 | 0 | 0 | 0 |
| Grade 1 | 0 | 0 | 0 | 0 | 0 | 0 |
| Grade 1-4 (total) | 3 (25%) | 6 (21%) | 7 (47%) | 6 (35%) | 4 (16%) | 26 (27%) |
| **Lipase (N)** | **12** | **28** | **15** | **17** | **25** | **97** |
| Grade 1 | 0 | 0 | 0 | 2 (12%) | 1 (4%) | 3 (3%) |
| Grade 2 | 0 | 1 (4%) | 0 | 2 (12%) | 0 | 3 (3%) |
| Grade 3 | 0 | 0 | 0 | 0 | 0 | 0 |
| Grade 1 | 0 | 0 | 0 | 0 | 0 | 0 |
| Grade 1-4 (total) | 0 | 1 (4%) | 0 | 4 (24%) | 1 (4%) | 6 (6%) |
| **Haemoglobin (N)** | **11** | **31** | **17** | **21** | **32** | **112** |
| Grade 1 | 1 (9%) | 2 (6%) | 0 | 2 (10%) | 1 (3%) | 6 (5%) |
| Grade 2 | 0 | 2 (6%) | 0 | 0 | 1 (3%) | 3 (3%) |
| Grade 3 | 0 | 1 (3%) | 0 | 0 | 0 | 1 (1%) |
| Grade 1 | 0 | 0 | 0 | 0 | 0 | 0 |
| Grade 1-4 (total) | 1 (9%) | 5 (16%) | 0 | 2 (10%) | 2 (6%) | 10 (9%) |
| **Neutrophils (N)** | **11** | **31** | **17** | **21** | **32** | **112** |
| Grade 1 | 0 | 1 (3%) | 1 (6%) | 2 (10%) | 2 (6%) | 6 (5%) |
| Grade 2 | 0 | 0 | 0 | 0 | 0 | 0 |
| Grade 3 | 1 (9%) | 1 (3%) | 1 (6%) | 0 | 0 | 3 (3%) |
| Grade 1 | 0 | 0 | 0 | 0 | 0 | 0 |
| Grade 1-4 (total) | 1 (9%) | 2 (6%) | 2 (12%) | 2 (10%) | 2 (6%) | 9 (8%) |
| **Platelets (N)** | **11** | **31** | **17** | **21** | **32** | **112** |
| Grade 1 | 0 | 0 | 1 (6%) | 0 | 0 | 1 (1%) |
| Grade 2 | 0 | 1 (3%) | 0 | 0 | 0 | 1 (1%) |
| Grade 3 | 0 | 0 | 0 | 0 | 0 | 0 |
| Grade 1 | 0 | 0 | 0 | 0 | 0 | 0 |
| Grade 1-4 (total) | 0 | 1 (3%) | 1 (6%) | 0 | 0 | 2 (2%) |

**N**: total number of participants with a post-baseline result available for each analyte. Results are presented by current weight band and formulation; hence, participants may appear in more than one weight band and formulation, as explained in previous footnote. The highest post-baseline emergent laboratory anomaly is reported by current weight band and formulation at time of the event. For example, a participant in the 14-<20 kg weight band who has two creatinine results of grades 1 and 2 (baseline grade 0), will be counted as one emergent grade 2 creatinine anomaly in that weight band and formulation, as that is the highest emergent grade for that analyte. A post-baseline analyte result is classed as emergent laboratory anomaly if grade increased from baseline. There were no emergent laboratory anomalies for white blood cell counts in the safety reporting period.

Table S7. Table with the detection range for DTG and 3TC, the within-run and between-run precision reported as coefficient of variation, and the accuracy.

|  | | **Conc.** | **Within run (n=5)** | | **Between run (n=15)** | |
| --- | --- | --- | --- | --- | --- | --- |
|  |  |  | *Accuracy* | *Precision* | *Accuracy* | *Precision* |
|  |  | *(mg/L)* | *(%)* | *(%)* | *(%)* | *(%)* |
| **Dolutegravir** | LLOQ | 0.01 | 100.94 | 4.66 | 102.11 | 2.56 |
|  | HLOQ | 20.0 | 99.92 | 2.70 | 100.86 | 0.96 |
| **Lamivudine** | LLOQ | 0.005 | 96.54 | 9.77 | 100.78 | 4.03 |
|  | HLOQ | 10.0 | 100.05 | 3.75 | 99.97 | 0 |

LLOQ, lower limit of quantification; HLOQ, higher limit of quantification.

Figure S1. DTG median concentration time profile by weight band, with the median shown as the central point and error bars representing the 25th and 75th percentiles to illustrate interindividual variability.


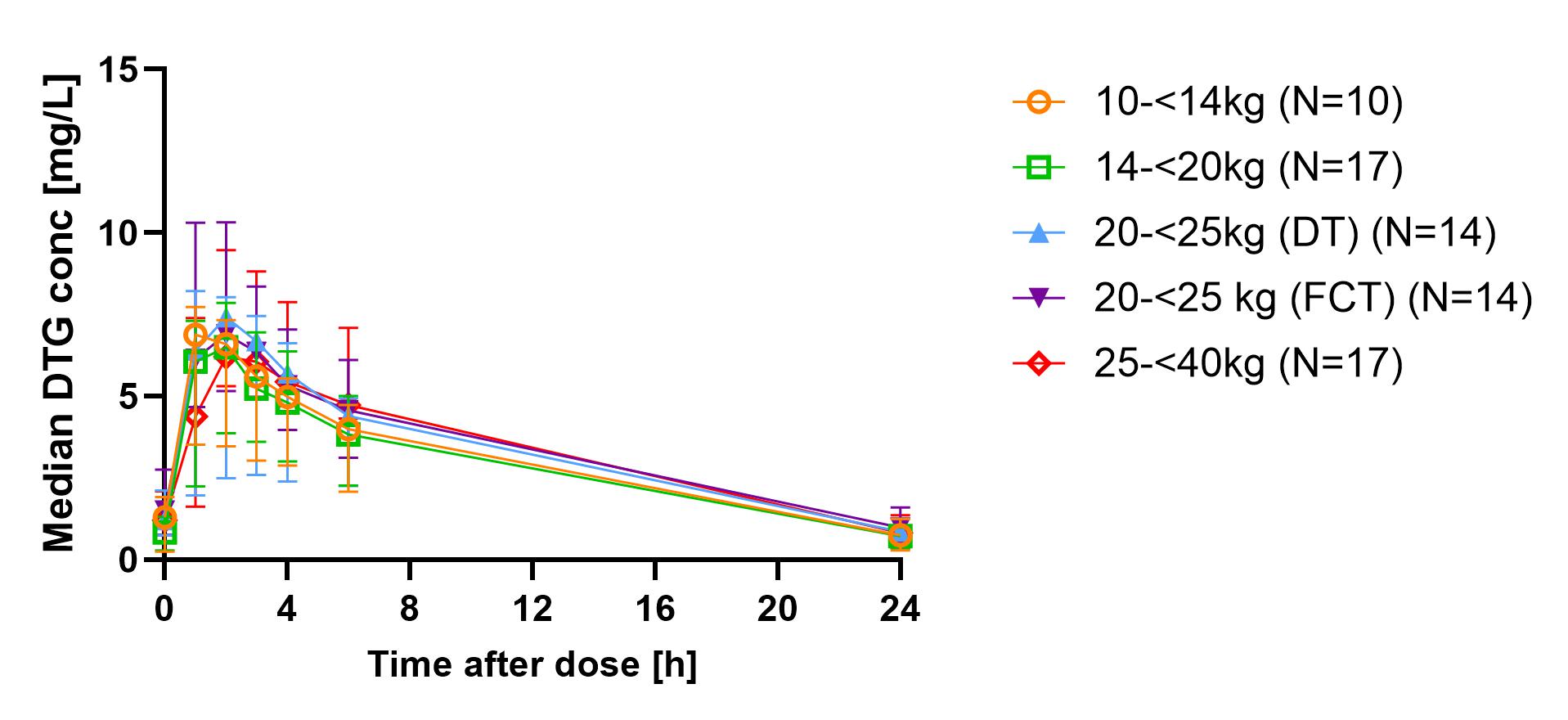


Figure S2. 3TC median concentration time profile by weight band, with the median shown as the central point and error bars representing the 25th and 75th percentiles to illustrate interindividual variability.


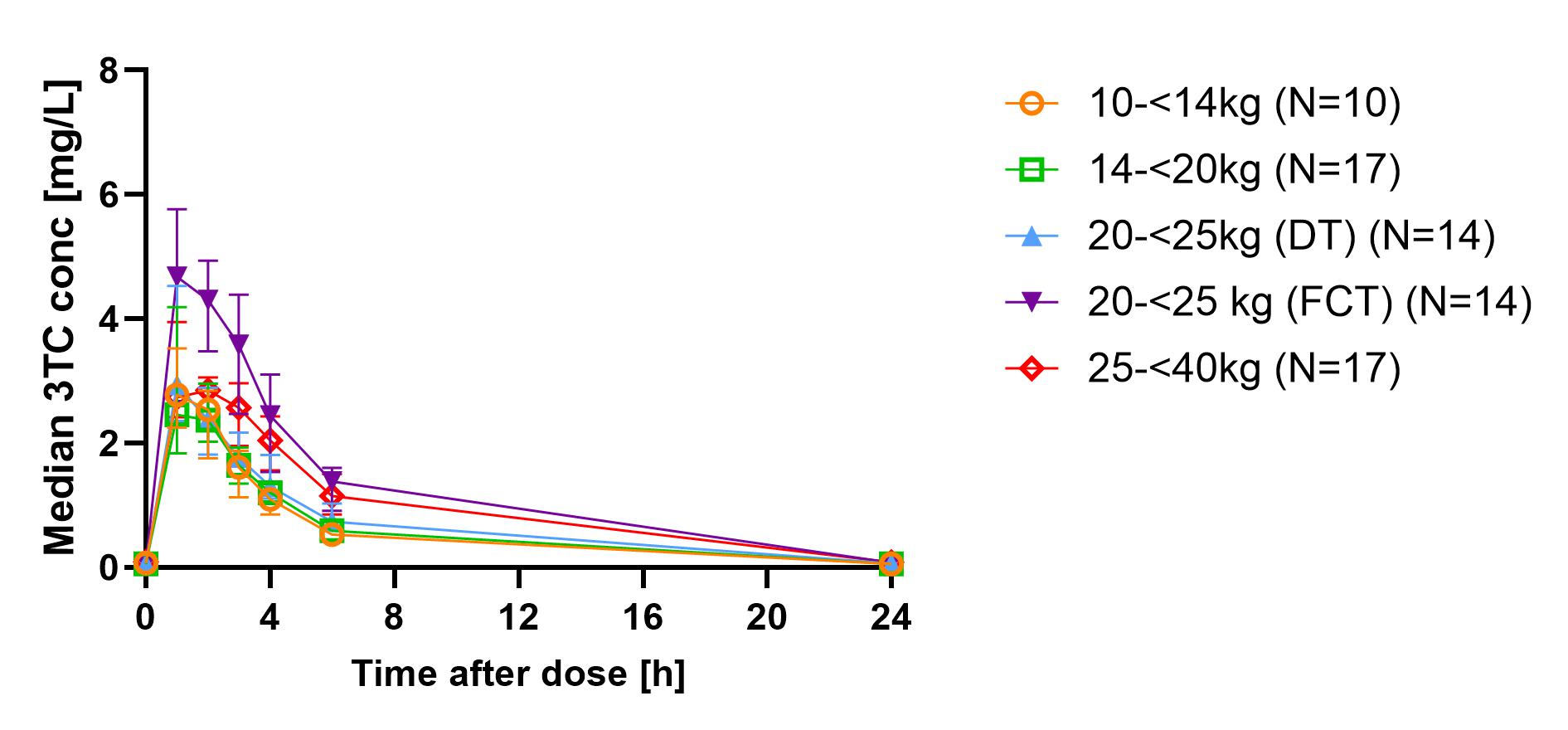


Figure S3. Line plots showing concentration kinetics for each participant, stratified by weight band group and analyte. The plots include all individual curves, even those that were excluded from the main analysis due to implausibility, to provide a comprehensive overview of the data.


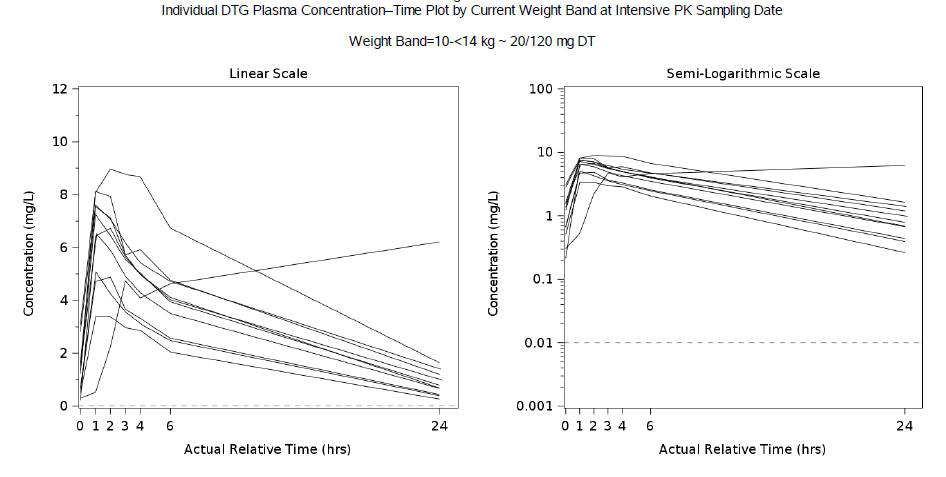


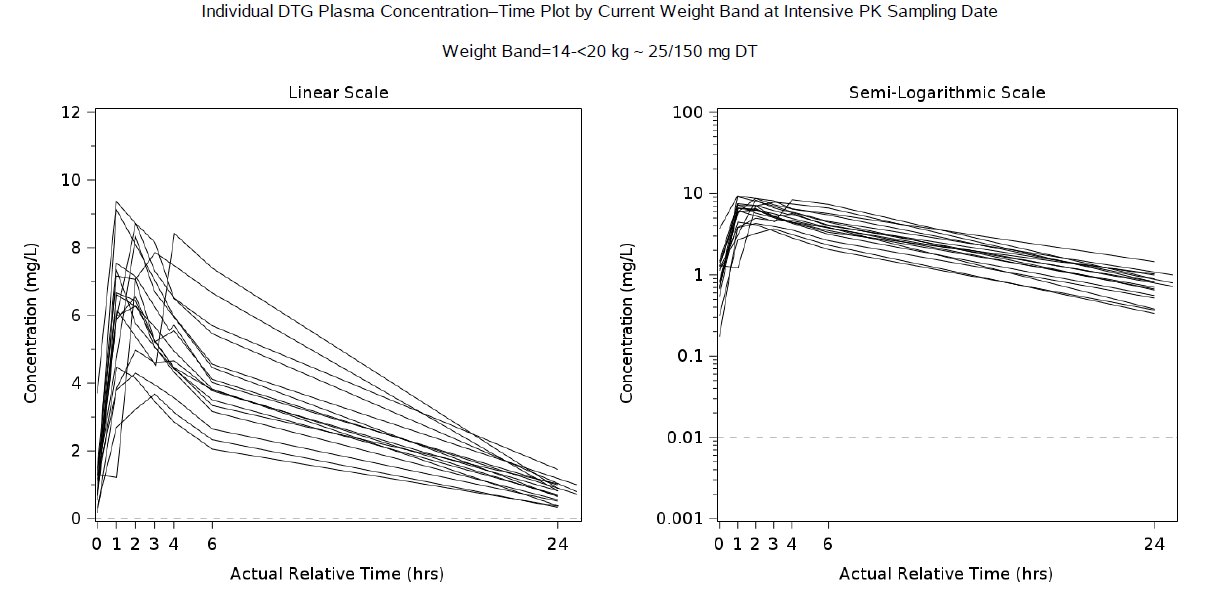


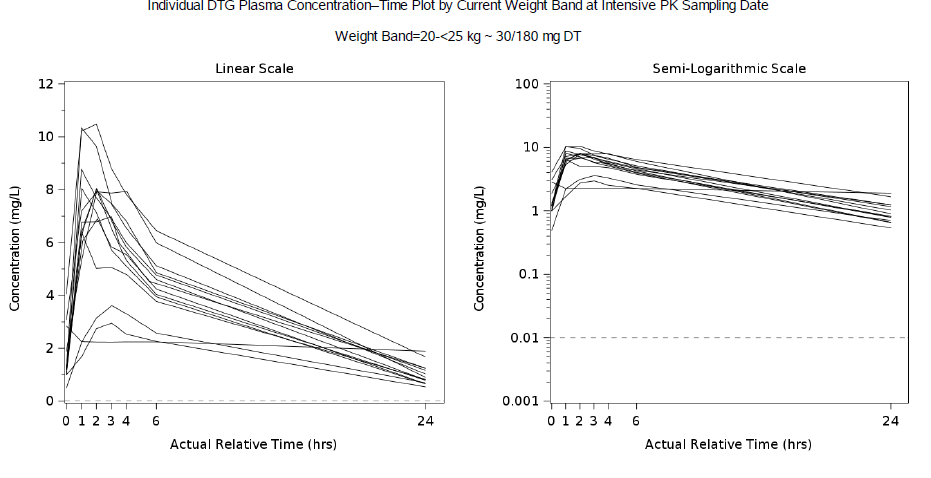


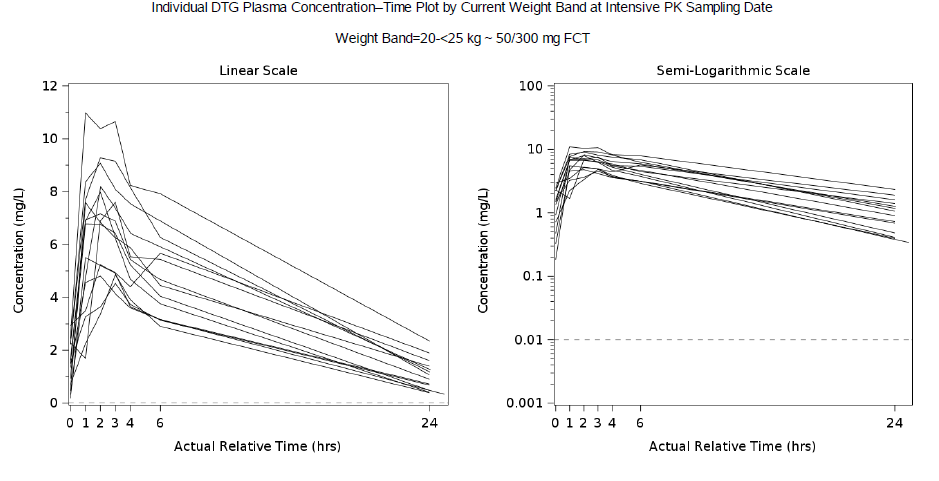


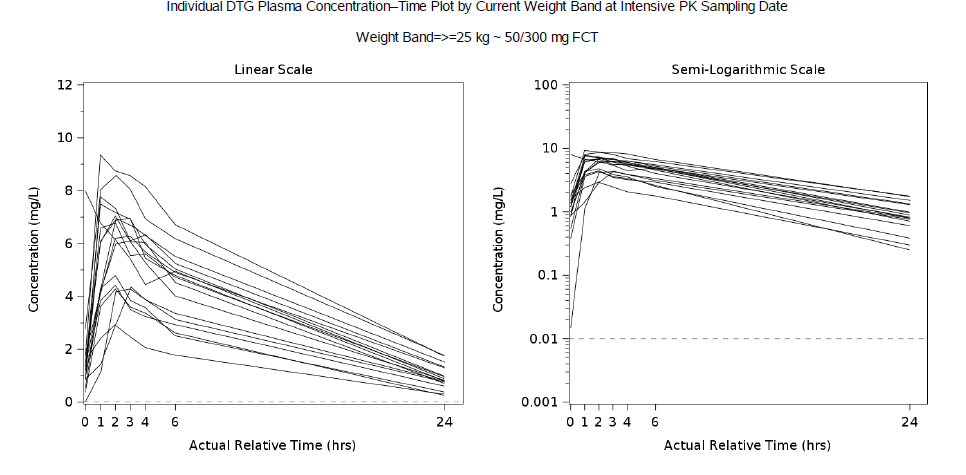


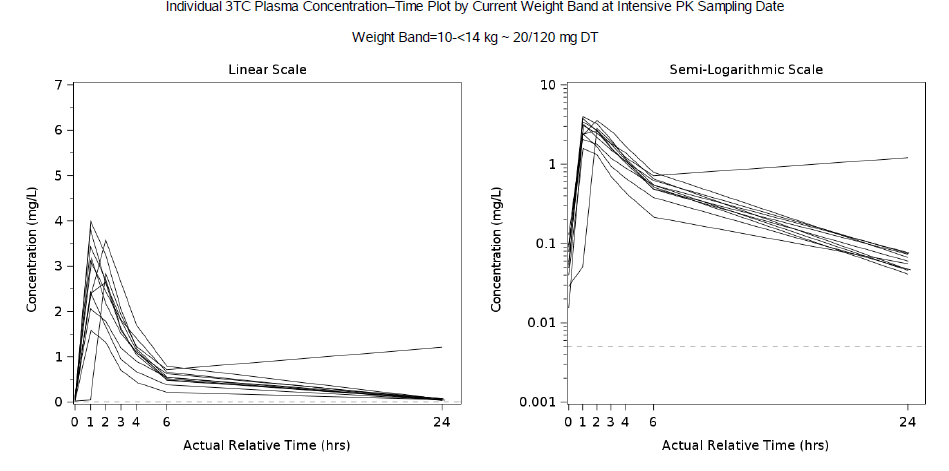


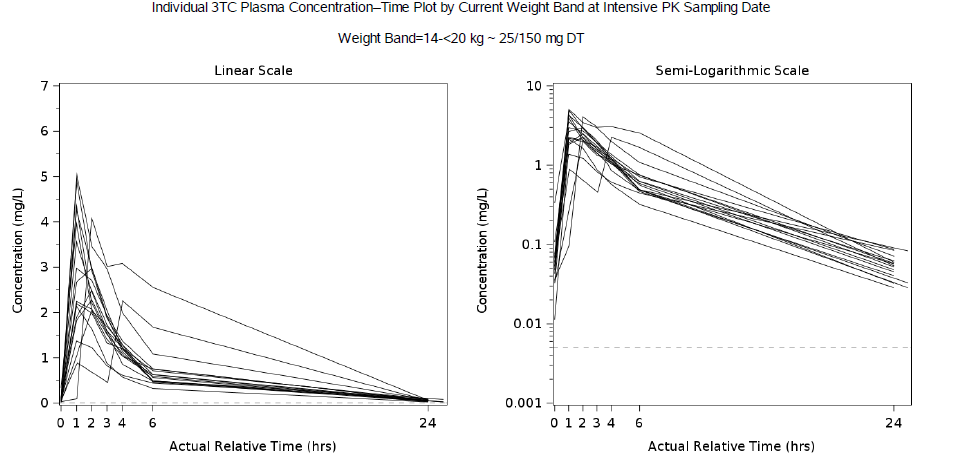


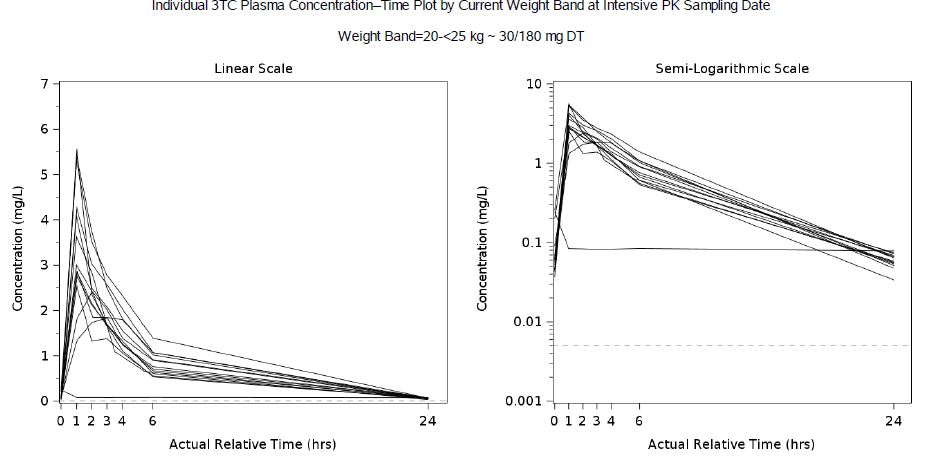


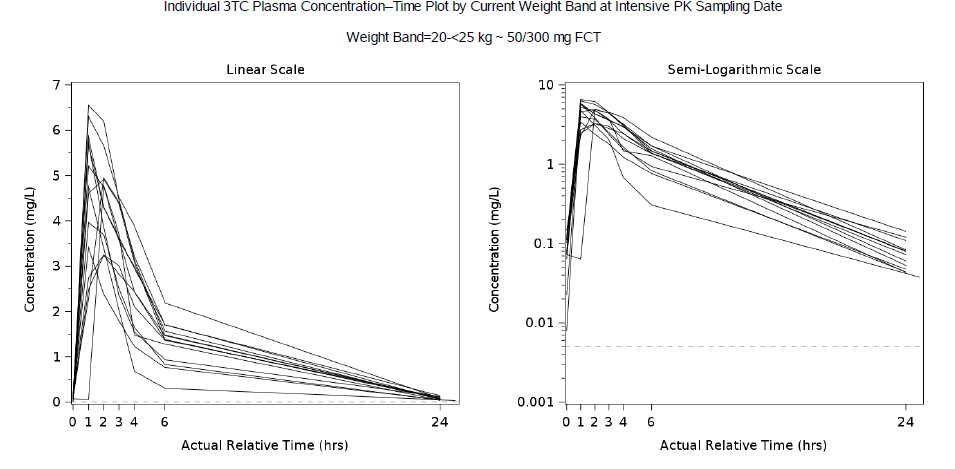


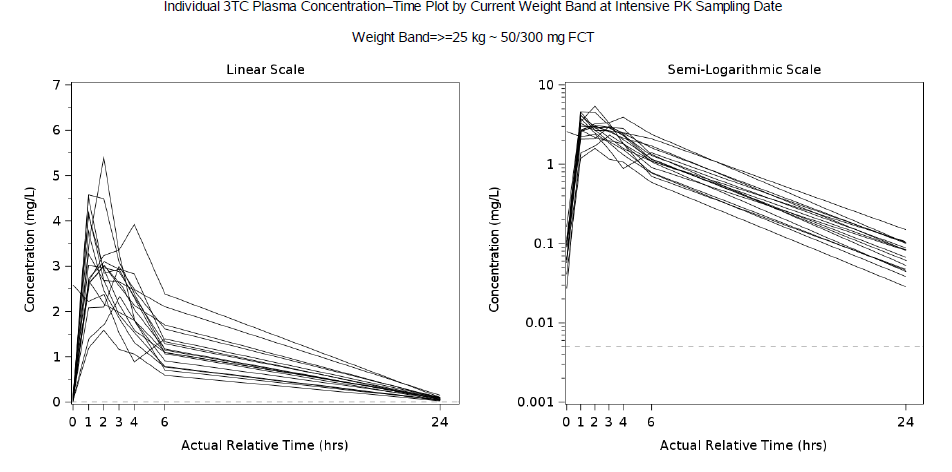

Supplement: Supplementary Material (pg no: 30–46) [file mmc2.docx]
